# Using Household Surveys to Assess Missed Opportunities for Simultaneous Vaccination: Longitudinal Examples from Colombia and Nigeria

**DOI:** 10.3390/vaccines9070795

**Published:** 2021-07-16

**Authors:** Dale A. Rhoda, Mary L. Prier, Caitlin B. Clary, Mary Kay Trimner, Martha Velandia-Gonzalez, M. Carolina Danovaro-Holliday, Felicity T. Cutts

**Affiliations:** 1 Biostat Global Consulting, Worthington, OH 43085, USA; Mary.Prier@biostatglobal.com (M.L.P.); Caitlin.Clary@biostatglobal.com (C.B.C.); MaryKay.Trimner@biostatglobal.com (M.K.T.); 2 Comprehensive Family Immunization Unit, Pan American Health Organization, Washington, DC 20037, USA; velandiam@paho.org; 3 Department of Immunization, Vaccines and Biologicals, World Health Organization, 1211 Geneva, Switzerland; danovaroc@who.int; 4 Department of Infectious Disease Epidemiology, London School of Hygiene and Tropical Medicine, London WC1E 7HT, UK; Felicity.Cutts@lshtm.ac.uk

**Keywords:** missed opportunities for vaccination, Vaccination Coverage Surveys, Demographic and Health Surveys, Nigeria, Colombia, Vaccination Coverage Quality Indicators, immunization

## Abstract

One important strategy to increase vaccination coverage is to minimize missed opportunities for vaccination. Missed opportunities for simultaneous vaccination (MOSV) occur when a child receives one or more vaccines but not all those for which they are eligible at a given visit. Household surveys that record children’s vaccination dates can be used to quantify occurrence of MOSVs and their impact on achievable vaccination coverage. We recently automated some MOSV analyses in the World Health Organization’s freely available software: Vaccination Coverage Quality Indicators (VCQI) making it straightforward to study MOSVs for any Demographic & Health Survey (DHS), Multi-Indicator Cluster Survey (MICS), or Expanded Programme on Immunization (EPI) survey. This paper uses VCQI to analyze MOSVs for basic vaccine doses among children aged 12–23 months in four rounds of DHS in Colombia (1995, 2000, 2005, and 2010) and five rounds of DHS in Nigeria (1999, 2003, 2008, 2013, and 2018). Outcomes include percent of vaccination visits MOSVs occurred, percent of children who experienced MOSVs, percent of MOSVs that remained uncorrected (that is, the missed vaccine had still not been received at the time of the survey), and the distribution of time-to-correction for children who received the MOSV dose at a later visit.

## 1. Introduction

A missed opportunity for vaccination (MOV) occurs when a person who is eligible for vaccination (with no true contra-indications) has contact with health services but does not receive all of their needed vaccines [1,2]. MOVs are commonly observed when a person attends health services for curative care due to inadequate integration of curative and preventive services. They may also occur when a person attends for vaccination. If they receive some but not all vaccines for which they are eligible, we use the term missed opportunity for simultaneous vaccination (MOSV). For example, a child who received a first dose of diphtheria-pertussis-tetanus-containing vaccine (DPTCV) at six weeks old but did not receive pneumococcal conjugate vaccine (PCV) on the same date, when the national schedule recommended both at the age of six weeks and no true contraindication existed, has a MOSV for PCV. MOVs are related to health system weaknesses such as vaccine stockouts; health worker failure to screen or to assess correctly which vaccines are due; misperceptions about contra-indications to vaccines; reluctance to administer multiple vaccines simultaneously or to open a vial for only one or two children; false beliefs of upper age limits; or health facility scheduling of different days for different vaccines [2,3,4,5]. It has long been recognized that health systems should prevent MOVs to maximize vaccination coverage and minimize susceptibility to disease [6,7], and reducing them is a key area of focus in the life course approach and integration strategy of the Immunization Agenda 2030 [8,9].

MOVs have most often been assessed by studies based at health facilities [2,10,11,12]. Such studies have the advantage of allowing investigation of the causes of MOVs but are limited in their generalizability [1,13]. By contrast, MOSVs can be assessed by appropriate analyses of household surveys that collect information on dates of each vaccine received, allowing estimation of their impact at the population level on coverage and on median age at vaccination [14,15,16,17,18,19,20,21].

In 2015, the World Health Organization (WHO) commissioned a set of specifications for immunization system performance indicators that can be calculated using vaccination coverage survey data. The resulting freely available documents and software are called the Vaccination Coverage Quality Indicators (VCQI (pronounced “Vicki”)) and can be calculated with any dataset that records children’s date of birth and dates of vaccination [20,22,23]. In this paper, we present results on trends in MOSVs in Colombia, a country with high coverage and no endemic measles transmission, and Nigeria, a country with low overall vaccine coverage and endemic measles transmission. We describe how VCQI’s MOSV indicators can serve as one form of standardized analysis to look for meaningful differences in MOSV prevalence across space and time and show examples of different ways to present results on MOSVs and their implications for population protection from vaccine-preventable diseases (VPDs). We discuss some challenges in calculating and interpreting MOSVs and suggest directions for future work.

## 2. Methods

We ran VCQI on DHS data from Nigeria’s surveys in 1990, 1999, 2003, 2008, 2013, and 2018 and on Colombia’s surveys from 1995, 2000, 2005, and 2010. We selected these countries because of our personal interest and involvement there (DAR and FTC and MCDH are frequently involved in vaccination coverage survey design and analysis in Nigeria; MCDH used to support Monitoring and Evaluation (M and E) on immunization in the Americas and MVG currently supports immunization M and E in the Americas and was formerly the national EPI manager in Colombia) and since Nigeria and Colombia contrast in overall immunization performance and in proportion of respondents aged 12–23 m whose dates of vaccination were available from home-based records (HBRs, or informally, cards).

### 2.1. Sources of Data

DHS reports for Nigeria were downloaded from the DHS Program website [24,25,26,27,28,29,30]. Data for Nigeria were downloaded from the Integrated Public-Use Microdata Series (IPUMS) DHS website [31]. IPUMS provides datasets with consistent variable coding, variable names, and geographic stratification across DHS rounds and across countries with DHS surveys. This facilitates consistent analysis using a single set of programs with very few modifications to accommodate survey-to-survey changes. In this case the only substantive differences between Nigeria DHS datasets were the list of vaccine-doses considered (Table 1) and the fact that the 1990 survey employed not only northern and southern geographic regions, but also a central region which was included in the national analyses but excluded from northern vs. southern stratification. For later surveys it was possible to easily assign all respondents to either the northern or southern portion of the country.

Data for Colombia were not available through IPUMS, so DHS reports and data were downloaded from the DHS program website [24,32,33,34,35].

### 2.2. Data Analysis

For both Nigeria and Colombia, we conducted MOSV analyses for the subset of children (a) who were 12–23 months of age at the time of the survey, (b) for whom month and year of birth was recorded within specified ranges, and (c) who had at least one date of vaccination available on their card. The day of birth was present for most children. If missing, it was imputed from the date of a birth dose, or, if no birth doses were recorded, imputed as being the first day of the month. Vaccines that were reported by the caregiver as having been received, or that had only a tick mark or illegible date on the card were not included in the analysis, as it cannot be determined whether these were valid doses nor if opportunities to receive other vaccinations were present at that vaccination visit. All dose dates were used to identify opportunities for simultaneous vaccination. However, for consistency across surveys, MOSVs were only summarized for the eight original EPI vaccine-doses: Bacille Calmette-Guérin (BCG), Oral Polio Vaccine (OPV) 1–3, (DPTCV) 1–3, and Measles-containing vaccine (MCV) 1. The DHS data were converted to use variable names and coding schemes expected by VCQI [36] and were analyzed using VCQI control programs running Stata version 16 [37,38]. The Stata programs are available from the corresponding author upon request.

VCQI summarizes results in Excel tables and Stata figures, and produces output datasets that may be further analyzed and mapped using other software. A publicly available R Shiny application was developed and used to visualize results of the child-based analyses [39,40,41].

## 3. Definitions

The WHO coverage survey manual recommends analyzing the number of health facility vaccination visits where there was at least one MOSV, and the number of children who experienced at least one MOSV [42]. We briefly describe the indicators here. Details are provided in [42] and details of VCQI calculations are described in the supplementary material.

### 3.1. Visit-Based Analyses

The visit-based (VB) analysis consists of three calculations that have the number of visits where a child was eligible to receive the considered dose(s) as the denominator: the proportion of visits resulting in a MOSV for a given vaccine (VB1), the proportion of visits resulting in at least one MOSV for any vaccine (VB2), and the average number of MOSVs per visit (VB3) (i.e., total number of MOSVs divided by the number of vaccine-eligible visits), and its inverse, which is interpreted as the number of vaccination visits between MOSVs (1/VB3).

### 3.2. Child-Based Analyses

The child-based (CB) analysis consists of two calculations: the proportion of children who had at least one MOSV for a given vaccine (CB1), and the proportion of children with at least one MOSV across all vaccines (CB2). CB1 can be further subdivided into the proportion of children who never received the particular vaccine (an uncorrected MOSV), and those who did receive it by the time of the survey (a corrected MOSV). Similarly, CB2 can be subdivided into the proportion of children where none, all, or some of the MOSVs for the child were corrected by the time of the survey.

After the visit and child-based MOSV analyses are conducted, it is possible to calculate the potential consequences of the MOSVs. First, the potential coverage that could have been achieved if there had been no missed opportunities is calculated. This is done by re-estimating coverage while giving credit for every dose that the child would have been age eligible for at each of their vaccination visits (assuming they had also received every eligible dose at every previous visit). Secondly, for children who had a corrected MOSV, the time to correction or delay (in days, weeks, or months) from the first opportunity until they finally received the vaccine can be calculated.

As described in Annex O of the WHO coverage survey reference manual [42], an MOSV analysis could be approached in two ways: (1) ignoring the age at receipt of vaccine or interval between doses so that any child with a date of vaccination is counted as vaccinated (described here and in the VCQI documentation as a “crude” dose analysis); or (2) treating doses given too early or after too short an interval as invalid (referred to as “valid” dose analysis). Minimum age at vaccination and interval between doses were defined as per the WHO summary tables of vaccination schedule recommendations [43,44]. If early doses are considered invalid, later visits would have potentially offered a chance to correct for the invalid dose by repeating it. For example, if DPTCV1 is scheduled to be given at six weeks of age, a child who received the first documented dose at four weeks of age would be discounted from the analysis of coverage according to valid doses [42,45]. If the child had received DPTCV2 it would count as DPTCV1, while DPTCV3 would count as DPTCV2. There may have been an opportunity to compensate for the invalid DPTCV1 doses prior to the actual date of DPTCV2, and there may have been an opportunity to give an additional dose at an older age (for example, at the time of the measles vaccination), which would mean the child could have had three valid doses. The difference between these approaches is illustrated in the Supplementary Materials. To our knowledge, previous studies of MOVs have ignored the potential to correct previous mistakes in following the recommended vaccination schedule. However, both approaches are presented here to illustrate the concept.

## 4. Results

### 4.1. Background Information on the Survey Populations

Table 1 lists the number of children aged 12–23 months in the DHS datasets and those included in the MOSV analysis. The doses summarized in the DHS reports changed over the years (e.g., transitioning from DPT to Pentavalent DPT-hepatitis-B-*Haemophilus influenzae* type-b vaccine (“penta”) in both countries and from measles to measles-mumps-rubella (MMR) vaccine in Colombia). In order to allow comparisons, only the original EPI vaccine-doses were analyzed, for which national recommended schedules are shown in Supplementary Tables SC.1 and SN.1. In later surveys, DPT coverage also represents that for *Haemophilus influenzae* type b (Hib) and Hepatitis B after the countries replaced DPT with pentavalent DPT-Hib-HepB. It was not considered practical to attempt to measure MOSVs for those vaccines during the time when they were administered as univalent vaccines. Colombia consistently had high proportions of respondents who showed cards to the interviewers, so more than half the children aged 12–23 m were included in the MOSV analyses there. Nigeria’s datasets have 20–40% with cards, meaning that the MOSV analysis is documenting the experience of less than half of those cohorts.

### 4.2. MOSV, Visit-Based Analyses

**Figure 1:**
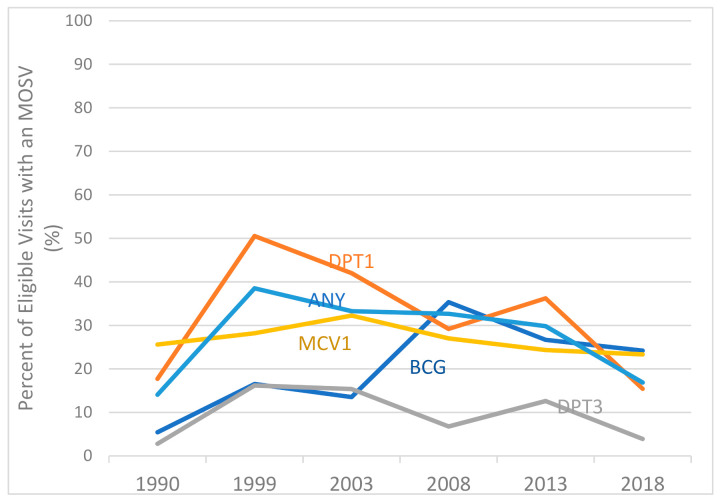
Percentage of eligible vaccination visits with a MOSV for BCG, DPT, MCV or any of the basic EPI vaccines, children aged 12–23 months, Nigeria 1990–2018.

Supplementary Tables SN.2a–c and SC.2a–c show the proportion of vaccine-eligible visits where MOSVs occurred, by vaccine-dose, and for any dose (of the traditional vaccines included in the analysis). Figure 1 shows dose-specific data for Nigeria over time (for clarity we omit OPV from the figure, but details are in the supplement). MOSVs occurred in up to 50% of visits where at least one vaccine was administered. Their frequency was approximately stable from 1999–2013. Frequency of MOSVs for DPT decreased sharply in 2018, while those for BCG and MCV1 decreased less dramatically. Interestingly, MOSVs were of similar frequency per visit in the north and south of the country except for MCV which is more frequent in the north (and therefore, “any dose MOSVs” are more frequent in the north) (Supplementary Tables SN.2a–c).

**Figure 2:**
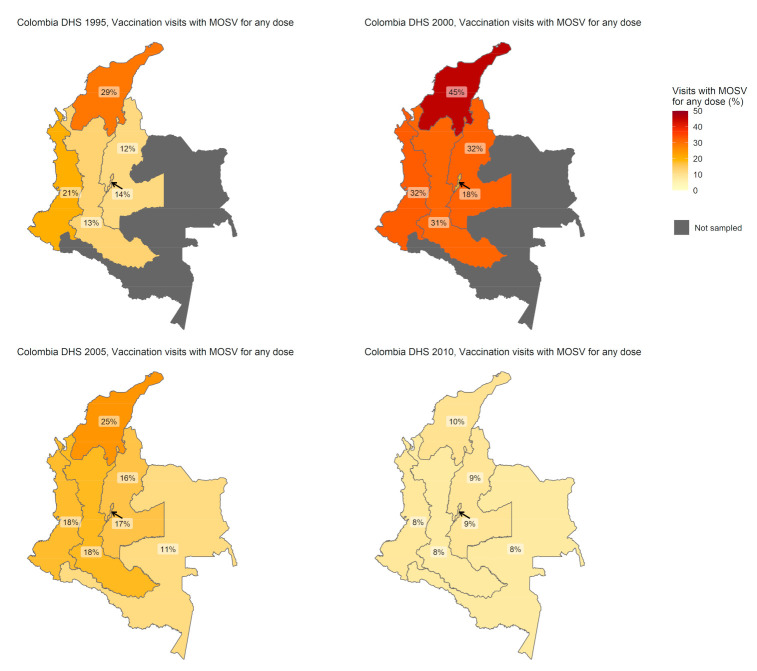
Percent of vaccination visits with one or more MOSVs, Colombia, 1995–2010. Map disclaimer: The boundaries and names shown, and the designations used on this map do not imply the expression of any opinion whatsoever on the part of the World Health Organization concerning the legal status of any country, territory, city or area or of its authorities or concerning the delimitation of its frontiers or boundaries. Dotted and dashed lines on maps represent approximate border lines for which there may not yet be full agreement.

For Colombia, Figure 2 presents maps of the percent of vaccine eligible visits with at least one MOSV over time. Results in 2000 showed a higher proportion of visits with MOSVs than in 1995 with MOSVs being most common in the Atlántica region in both the early surveys. Performance improved noticeably by 2005, and by 2010 the performance was uniformly good with MOSVs in fewer than 10% of vaccination visits in every region. (The electronic supplement includes a map with region names, Map SC.1, and additional details in Tables SC.2a–c).

### 4.3. MOSV, Child-Based Analyses

**Figure 3:**
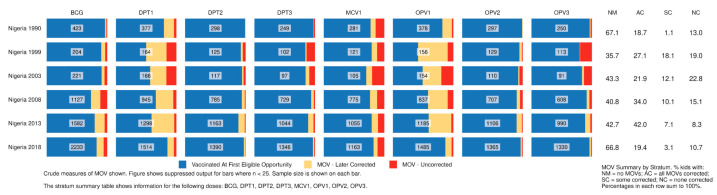
Proportion of children who experienced MOSVs & correction status at the time of survey, by dose, Nigeria 1990–2018. Detailed counts of children represented in each Figure 3 bar portion are documented in supplementary Table SN.3a.

**Figure 4:**
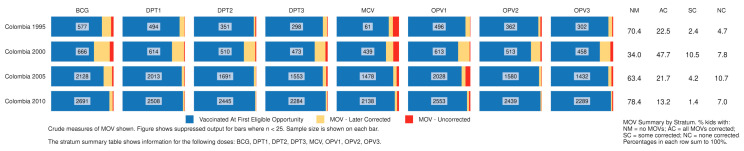
Proportion of children who experienced MOSVs & correction status at the time of survey, by dose, Colombia 1995–2010. Detailed counts of children represented in each Figure 4 bar portion are documented in supplementary Table SC.3a.

Figure 3 and Figure 4 show output from the R Shiny tool that summarizes child-based MOSV outcomes for Nigeria and Colombia, respectively. Each small bar represents 100% of children in the sample from that geographic stratum who showed interviewers a card and had at least one vaccination visit when they were eligible for the dose. The sample size appears in the center of each bar. The blue portion represents children who were vaccinated at their first eligible visit. Yellow and red represent corrected and uncorrected MOSVs, respectively. Tabular information at the far right documents the proportion who experienced at least one MOSV for any dose and what portion had all or some of their MOSVs corrected by the time of the survey.

For Nigeria, Figure 3 shows that almost two-thirds of children in the analysis had at least one MOSV in DHS surveys from 1999–2013, but this fell to one-third in 2018. The proportion of corrected MOSVs increased until 2013 when 42% of children analyzed received all the missed doses by the time of the survey. In 2018, however, 11% of children had MOSVs with none corrected and 63% of children who had a MOSV for MCV1 had not received the vaccine by the time of the survey (128/203; detail in supplementary Table SN.3a). The electronic supplement shows results for the north versus south indicating that a higher proportion of children sampled from the north experience MOSVs for MCV1, OPV1 and DPT1 than in the south but the portion of MOSVs that are corrected are quite similar (Supplementary Figure SN.3 and Table SN.3b).

In Colombia, Figure 4 shows that apart from 2000, the proportion with at least one MOSV was between 22 and 37% and most MOSVs were corrected. Encouragingly, there were very few MOSVs for MCV in the later surveys (details and subnational results appear in supplementary Figure SC.3 and Tables SC.3a and SC.3b).

### 4.4. Potential Consequences

**Figure 5:**
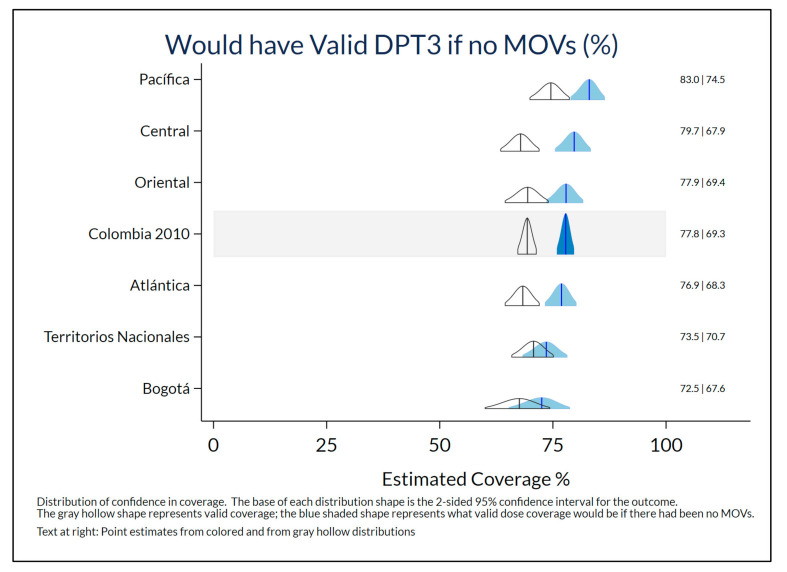
Observed valid coverage of DPT3 and potential valid coverage achievable if every dose due had been administered at every vaccination visit, among children aged 12–23 months with HBRs, Colombia DHS 2010.

This measure of MOSV potential consequence is most meaningful in studies with high card availability. The weighted estimate of cards seen was 83.2% in Colombia in 2010, hence the results for that year are presented here. Figure 5 indicates that estimated valid dose coverage for DPT3 would have been 8.5% higher in Colombia in 2010 if the children in the survey had received DPT appropriately at every eligible visit with no early doses and no MOSVs. (Early doses represent about 3/8 ths of the 8.5% possible improvement.) The observed estimate of valid coverage was 69.3% instead of the counterfactual, but maybe achievable 77.8%. The highest difference was in the Central region (11.8%) and smallest in Territorios Nacionales (2.8%). (National results for other doses appear in Supplementary Table SC.4. Nigeria’s results appear in Table SN.4.)

### 4.5. Delay to Receipt of Missed Doses

**Figure 6:**
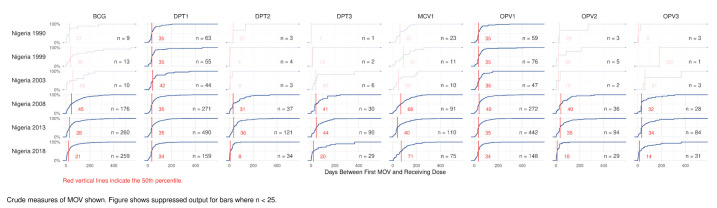
Cumulative distribution of time to MOSV correction, in days, by dose, Nigeria 1990–2018. Additional quantiles for Nigeria are documented in supplementary Table SN.5a and subnational detail is in Table SN.5b.

**Figure 7:**
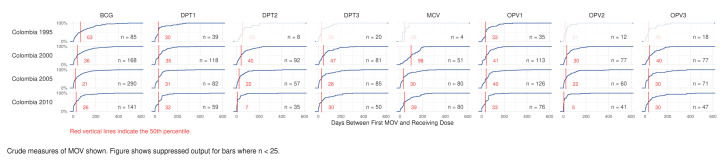
Cumulative distribution of time to MOSV correction, in days, by dose, Colombia, 1995–2010. Additional quantiles for Colombia are documented in supplementary Table SC.5a and subnational detail is in Table SC.5b.

Another measure of MOSV consequence is to quantify the number of extra days when children are un- or under-protected from VPDs because of missed opportunities. Figure 6 and Figure 7 show national output from the R Shiny tool that summarizes child-level results. Each block shows the cumulative distribution of the number of days between the original MOSV and the date when it was corrected. The horizontal axis shows time to correction from 0 to 600 days. The vertical axis represents the cumulative percentage, from 0 to 100%, of the children whose MOSVs were corrected by the number of days lapsed since the earliest opportunity for vaccination, up to the time of the survey. The sample size is indicated in each block. Blocks with sample sizes smaller than 25 are intentionally made opaque. The median delay appears with a vertical red line and red number. Figure S.4 provides additional orientation to the time to correction plots. The tool can also show the 25th, 75th, and 90th percentiles. Those are tabulated in Tables SN.5a,b and SC.5a,b. In Nigeria, the median time to correction is consistently just over one month for doses scheduled in early infancy and somewhat longer for MCV1. From 2013 to 2018 the median delay dropped by at least 20 days for both OPV3 and DPT3, although the number of corrected MOSVs in the 2018 dataset was notably much smaller than in 2013. Conversely, the median delay for MCV1 increased from 40 to 71 days. Colombia’s median delays are in a range similar to those observed in Nigeria.

## 5. Discussion

Missed opportunities are an important cause of incomplete and delayed vaccination coverage [2,46]. They also highlight the importance of having quality home-based and facility-based immunization records which health workers can review in each contact with a child [47]. Past studies have shown that if a mother takes a child to a clinic for vaccination but does not receive the scheduled vaccine, she may be deterred from returning [15,48]. Hence, MOVs are a barrier to using available health services. Even if mothers return, the delay before the child is protected increases the risk of VPDs. During an era when health services are increasingly stretched by the direct and indirect effects of the Covid-19 pandemic, it is vital to use all opportunities to vaccinate children at every contact with health facilities and to remember that it is better to vaccinate late than never [3].

The freely available WHO VCQI software assesses missed opportunities for simultaneous vaccination using three well-established indicators of frequency at each vaccination visit, frequency per child, and consequences in terms of delay until vaccination or overall coverage achieved. VCQI can be used with archived survey datasets so is suitable to assess changes in MOSV frequencies and consequences over time and space. We have shown how such analyses provide insights into program performance in two contrasting settings.

Supplementary Figure SVPD.1 shows reported incidence rate data for five VPDs over the past forty years. Except for a pertussis outbreak in the 2010s, Colombia’s rates for all five diseases have been nearly zero for more than twenty years. Nigeria’s reported rates have dropped substantially since 2000 and are approaching those of Colombia with the recent exception of diphtheria and periodic challenges with measles. Note that these figures represent cases of VPDs reported to WHO and likely underestimate true incidence.

In Nigeria, MOSVs were least frequent in the 1990 DHS (occurring in 14% of 1351 occasions where at least one vaccine was documented to have been received) and most frequent in 1999 (occurring in 38.5% of 732 documented visits, supplementary Table SN.2c); in subsequent years they occurred in 30–33% of visits until a sharp drop to 16% in 2018. In general, MOSVs were most common for the first dose of OPV or DPT and for MCV but those for BCG increased markedly from 2008, perhaps suggesting a shortage of BCG in health facilities that administer the other vaccines, fewer health workers skilled in intradermal injections needed for BCG or health worker hesitancy to open a multidose BCG vial for fear of high wastage rates. Although the proportion of MOSVs that were corrected increased over time, a substantial proportion of missed vaccine-doses were never administered. Often, there is no formal system for identifying doses that were given early and re-administering them at later visits, so VCQI’s crude dose analysis is consistent with the way immunization programs work in most countries. VCQI can document the proportion of doses administered early or with intervals that are too short, and may be used to comment on the portion of children with cards who might be at risk of not developing an immune response because of inappropriate vaccination timing [37]. If policies were explored to readminister mistimed doses, then VCQI’s valid dose analysis could quantify the number of opportunities that exist to do that and can document when those opportunities are missed.

In Nigeria, the delay between the first opportunity to be vaccinated and receipt of the vaccine was substantial and especially important for MCV with a delay of approximately 10 weeks in the 2018 DHS. Although the time to correction was similar in the north and south of Nigeria (supplementary Table SN.5b), this avoidable time at risk of measles is particularly concerning in northern Nigeria which is subject to frequent large outbreaks [49].

According to WHO/UNICEF estimates (2020 release), in 2019 Nigeria had the largest number of children in the world who had not received the first dose of DPTCV (approximately 2.5 million) or MCV (3.3 million) [50,51]. Offering all vaccines for which a child is eligible each time a child attends for vaccination should be one of the simplest interventions to increase coverage yet there has been little progress in reducing MOSVs for BCG and MCV to date. MOVs are even more frequent during health care contacts for other reasons such as curative care or accompanying a caregiver to a health facility [52]. Developing and implementing an effective plan to reduce MOVs in Nigeria is of very high priority and analysis of subsequent household surveys will facilitate monitoring the effectiveness of interventions.

The pattern of MOSVs seen in Colombia with the highest rates observed in the 2000 DHS may have been related to changes in the responsibilities related to the immunization program management, and the historic changes the country was experiencing. Colombia decentralized responsibilities and resources, including those for the immunization program, to subnational governments beginning in the late 1980s and regulated this decentralization reform through Law 60 (1993) and Law 715 (2001). In 1993, Colombia introduced “Law 100,” a health care reform package with the aim of improving access to care and provide financial protection to all the population in the country. Between 1991 and 1994, Colombia experienced important economic growth followed by a recession in the context of intensified armed conflict [53] in 1998–1999. Confusion about responsibilities for public health activities between different actors, fragmentation of resources and responsibilities in public health, and the time it took for the subnational government to gain experience in management capacity [54] were factors that could have contributed to the inadequate performance of the EPI in Colombia in the 1990s. Subsequently, the subnational governments adapted, likely leading to increased coverage and a decrease in MOSVs from 1995 to 2010. However, even in 2010 there was room for improvement. DPT3 coverage would have been 8.5% higher if children had received the vaccine at every eligible visit, and this is not even considering the long time it took to correct those missed opportunities that had been corrected by the time of the survey. Identifying the reasons for missed opportunities at the local level should be the next step.

The Pan American Health Organization (PAHO), an international public health agency for the people of the Americas and serving as the WHO regional office for the Americas, has guided countries on how to assess and reduce MOVs since 1980s. In 1988, PAHO adapted a protocol for health-facility studies prepared by WHO and demonstrated high MOV rates, ranging from 34–77% in 10 countries [55]. In 2013 the methodology was reviewed with the goal of increasing immunization coverage in vulnerable municipalities and PAHO made available a standardized methodology for evaluating MOVs in children aged <5 years in primary and secondary health facilities and for evaluating the vaccine-related attitudes and knowledge of health workers [11].

Interventions to reduce MOVs include improving health care workers’ skills to assess eligibility for a vaccine and improving both their knowledge regarding false contraindications and their soft skills to talk with guardians to better inform the importance of the vaccines [47]. At the global level, the WHO is leading a body of work to reduce MOVs, having updated the generic protocol to measure the prevalence and causes of MOVs at a sample of health facilities. The WHO strongly encourages countries to develop national strategies to reduce MOVs by measures such as better integration of services, improved logistics to prevent stockouts, ensuring the availability and effective use of home-based and facility-based records, providing vaccines daily especially in densely populated areas, in-service training and supportive supervision. Analysis of existing survey datasets is also encouraged to demonstrate the importance of MOVs and motivate Ministries of Health and their partners to prioritize the MOV reduction strategy.

VCQI is compatible with any coverage survey design that documents the date when a child was born and their dates of vaccination. It can also give useful output if only the month and year of birth is known by imputing the day of birth. It can be used to examine MOSVs in recent surveys that were conducted using WHO’s 2018 Vaccination Coverage Cluster Survey Reference Manual, as well as older EPI survey designs such as the classic 30 × 7 design [42,56]. It is flexible to accommodate any EPI vaccination schedule and currently handles one-, two-, and three-dose series. It could be upgraded to handle series with more doses. Since VCQI does not try to assess the underlying reasons for the MOSVs, it does not require interaction with children’s caregivers or health care providers, so it could be used to analyze datasets recorded solely from facility-based records or electronic immunization registries. Its results will be most accurate if the vaccination dates are complete and correct. If the child’s health record does include dates of non-vaccination visits, it is trivial to add those dates to the VCQI analysis and produce a more comprehensive picture [15,18]. However, it adds to the time needed for the interviewer to record all the evidence. An algorithm like VCQI’s could also serve as the basis for MOV analyses to be included into electronic immunization registries (EIRs) to facilitate decision-support. VCQI can also be used to analyze legacy data from EIRs, allowing more extensive comparison of MOSV rates at subnational levels.

As noted in a helpful 2014 review, MOVs have been assessed using a wide variety of approaches that bode well for creative insights but might be most broadly helpful if implemented in a tool that can be run (or re-run) on a broad array of archived datasets [13]. Because such datasets are archived and available from DHS and MICS [57], a tool such as VCQI is a good place to instantiate some of those indicators. Others have analyzed proportion of children who experience MOVs across a cross-section of archived DHS datasets [58,59], but we believe this is the first longitudinal analysis showing both visit-based and child-based indicators. VCQI, and its underlying algorithm, addresses the need that other researchers have recently noted in this journal for vaccine-specific and dose-specific indicators [60].

When only a subset of children in the survey show vaccination cards with dates, the indicator results may be indicative, but not fully representative, of vaccination program performance. Persons with cards seen in surveys differ from those without cards [61,62]. If the dates are either recorded with errors or transcribed with errors, the MOSV indicators will contain errors, too. MOSVs may be more or less likely among children without an HBR. On the one hand, children without cards may come from families who use health services less than those with a card and hence have fewer opportunities to be vaccinated. On the other hand, if a child without a card does attend a clinic, they may be more likely to experience MOSVs than those who faithfully take the card to the clinic, since it is difficult for the clinic worker to know which doses they should receive [47]. Thus, in populations such as Nigeria where a notable portion of respondents are missing documented vaccination dates, VCQI MOSV output may show better MOSV performance than the true average. Furthermore, recent studies show that missed vaccination opportunities are most common when children attend for curative care. Thus, VCQI results only assess one component of the health system performance for vaccination [63]. Nonetheless, even without formal statistical tests or high-precision confidence intervals, broad-stroke results based on survey respondents with documented dates may suffice to inspire collaboration to address MOVs. If the MOSV analysis of a coverage survey indicates that MOSVs are prevalent, then a follow-up clinic-based study can be indicated to identify all types of MOVs and determine their reasons [64,65,66,67]. The choice of health facilities to include in such a study can be informed by stratum-specific results from the household survey (if it is large enough). In countries such as Nigeria where it is clear that MOVs are a problem (from the analyses reported here and from in-depth work in Kano state) [52,68], the WHO MOV strategy suggests that country stakeholders might skip the clinic-based assessment and move directly to brainstorming about interventions [2,12,69,70].

This study has several strengths. It uses publicly available data and publicly available auditable Stata programs to conduct standard analyses according to definitions from the recently updated WHO reference manual [42]. The VCQI software eliminates nonsensical vaccination dates before identifying MOSVs, converting them to tick marks so the child receives credit for receiving the dose. However, dates that are obviously wrong (vaccination before birth, vaccination after the survey, vaccination series dates out-of-order or series with the same date for consecutive doses, etc.) do not enter the analysis. The analyses are consistent across time and countries, which has been a notable challenge [13]. The Nigeria analysis uses data from IPUMS which is coded consistently so data from five surveys spanning nearly twenty years can be analyzed using a single set of VCQI analysis parameters.

This study also has several limitations in addition to the likely bias related to representativeness due to availability of vaccination cards. Most of the reports for DHS surveys included here note that not all areas in the country were accessible. If those regions were difficult for survey interviewers to reach, they may have also been difficult for vaccine cards and supplies to reach. Thus, children there may have experienced even more MOVs than those who are included in the survey samples. While VCQI identifies and removes nonsensical dates, it assumes that dates that are not obviously wrong must be correct, but of course this is not always the case [71,72]. Finally, only selected vaccines and only a single year age cohort from each survey were analyzed. As countries add routine immunization doses in their schedules beyond the first dose of measles vaccine, it will be instructive to extend MOV analyses to include older cohorts to identify all the opportunities for MOSV corrections.

## 6. Conclusions

In short, a standardized assessment of missed opportunities for simultaneous vaccination is freely accessible to survey analysts using WHO’s VCQI software. Using the same type of data, we have shown that for two diverse countries, MOSVs were reduced over time. However, there is much that remains to be done. While MOSVs at vaccination visits are only a subset of all MOVs, the ability to assess survey datasets archived across space and time in a consistent manner is powerful. The source code for VCQI is freely available and designed to be extensible, so additional MOV indicators could be added either by WHO or by other analysts with Stata programming skills.

## Figures and Tables

**Table 1:** Surveys and doses and children available for analysis.

Country	Year	Vaccines Included in DHS Report	Age 12–23 m in DHS Dataset N	Card Seen N (%)	Included in MOSV Analysis * N (%)
Colombia	1995	BCG from birth DPT doses 1–3 OPV doses 1–3 MCV/Measles dose 1	1031	630 (61.1%)	615 (59.7%)
	2000	BCG from birth DPT doses 1–3 OPV doses 1–3 MCV/MMR dose 1	914	686 (75.1%)	683 (74.7%)
	2005		2919	2208 (75.6%)	2201 (75.4%)
	2010	BCG from birth Penta/DPT doses 1–3 OPV doses 1–3 MMR dose 1	3435	2895 (84.3%)	2891 (84.2%)
Nigeria	1999	BCG from birth DPT doses 1–3 OPV birth dose OPV doses 1–3 MCV/Measles dose 1	1042	217 (20.8%)	210 (20.2%)
	2003		987	232 (23.5%)	225 (22.8%)
	2008		5022	1269 (25.3%)	1243 (24.8%)
	2013		5834	1764 (30.2%)	1707 (29.3%)
	2018	BCG from birth HepB birth dose Penta doses 1–3 OPV birth dose OPV doses 1–3 IPV dose 1 PCV doses 1–3 MCV/Measles dose 1	6057	2426 (40.1%)	2271 (37.5%)

* Those with a card but not in MOSV analysis either lacked a valid date of birth or had no vaccination dates on the card.

## Data Availability

The datasets analyzed here are freely available to registered researchers at the Demographic and Health Studies website (https://dhsprogram.com/data/ accessed on 16 July 2021).

## Supplementary Materials

The following are available online at https://www.mdpi.com/article/10.3390/vaccines9070795/s1, Figure SVPD.1. Incidence rates for vaccine preventable diseases for Colombia & Nigeria, 1980–2020. Figure S.1. Vaccination Evidence and MOSV Indicators—Respondent 2—had MOSVs for Penta2 and Penta3. Text Box S.1. Features of an “Evidence and Indicators Plot”. Text Box S.2. Vaccination experience for children in Figures S.1, S.2, and S.3a,b. Figure S.2. Vaccination Evidence and MOSV Indicators—Respondent 3—had MOSVs for Penta1. Figure S.3a. Vaccination Evidence and MOSV Indicators—Respondent 4—VCQI crude dose analysis where early doses count. Figure S.3b. Vaccination Evidence and MOSV Indicators—Respondent 4—VCQI valid dose analysis where early doses do not count. Figure S.4. Time to Correction Figure—Interpretation Guide. Text Box S.4. Features of a Time to Correction Plot. Table SC.1. Vaccination schedule for doses in Colombia MOSV analysis. Table SN.1. Vaccination schedule for doses in Nigeria MOSV analysis. Map SC.1. Map of Colombia Showing Region Names. Table SN.2a. Visit-based MOSV analysis—Nigeria—BCG and OPV1-3. Table SN.2b. Visit-based MOSV analysis—Nigeria—DPT1-3 and MCV1 and any dose. Table SN.2c. Visit-based MOSV analysis—Nigeria—Any Dose, MOSVs per visit & Visits between MOSVs. Table SC.2a. Visit-based MOSV analysis—Colombia—BCG and OPV1-3. Table SC.2b. Visit-based MOSV analysis—Colombia—DPT1-3 and MCV1. Table SC.2c. Visit-based MOSV analysis—Colombia—Any dose, MOSVs per visit & Visits between MOSVs. Table SN.3a. Child-based MOSV analysis—Nigeria—National Results. Figure SN.3. Child-based MOSV analysis—Nigeria—Subnational Detail. Table SN.3b. Child-based MOSV analysis—Nigeria—Subnational Detail. Table SC.3a. Child-based MOSV analysis—Colombia—National Results. Figure SC.3. Child-based MOSV analysis—Colombia—Subnational Detail. Table SC.3b. Child-based MOSV analysis—Colombia—Subnational Detail. Table SN.4. MOSV Consequences—Nigeria—Potential Coverage Increase—National Results. Table SC.4. MOSV Consequences—Colombia—Potential Coverage Increase—National Results. Table SN.5a. MOSV Consequences—Nigeria—Time to Correction—National Results. Table SN.5b. MOSV Consequences—Nigeria—Time to Correction—Subnational Detail. Table SC.5a. MOSV Consequences—Colombia—Potential Coverage Increase—National Results. Table SC.5b. MOSV Consequences—Colombia—Potential Coverage Increase—Subnational Detail. The supplement also contains a description of the provenance of VCQI MOSV indicators and of data quality complications and calculation complications.
