# Supplementary material for: Using Household Surveys to Assess Missed Opportunities for Simultaneous Vaccination: Longitudinal Examples from Colombia and Nigeria
Source: Vaccines (Basel). 2021 Jul 16;9(7):795. doi: 10.3390/vaccines9070795 (PMC8310031; doi:10.3390/vaccines9070795)
Supplement: Supplementary file 1 [file vaccines-09-00795-s001.zip › vaccines-1267465-supplementary.pdf]

Supplementary Materials

Using Household Surveys to Assess Missed Opportunities for Simultaneous Vaccination: Longitudinal Examples from Colombia and Nigeria

Dale A. Rhoda[1*], Mary L. Prier[1], Caitlin B. Clary[1], Mary Kay Trimner[1], Martha Velandia-Gonzalez[2], M. Carolina Danovaro-Holliday[3], Felicity T. Cutts[4]

Table of Contents

Incidence of Vaccine Preventable Diseases 3
Provenance of the VCQI MOSV Indicators 4
Graphical Examination of Missed Opportunities 5
MOSV Data Quality Complications 13
MOSV Calculation Complications 13
Vaccination Schedules for MOSV Analyses 15
References 16
Additional Details to Supplement Figures in the Manuscript 17

List of Tables and Figures

Figure SVPD.1. Incidence rates for vaccine preventable diseases for Colombia & Nigeria, 1980-2020 3

Figure S.1. Vaccination Evidence and MOSV Indicators – Respondent 2 – had MOSVs for Penta2 and Penta3 6

Text Box S.1. Features of an "Evidence and Indicators Plot" 7

Text Box S.2. Vaccination experience for children in Figures S.1, S.2, and S.3a-b 8

Figure S.2. Vaccination Evidence and MOSV Indicators - Respondent 3 – had MOSVs for Penta1 9

Figure S.3a. Vaccination Evidence and MOSV Indicators - Respondent 4 – VCQI crude dose analysis where early doses count 10

Figure S.3b. Vaccination Evidence and MOSV Indicators - Respondent 4 – VCQI valid dose analysis where early doses do not count 11

Figure S.4. Time to Correction Figure – Interpretation Guide 12

Text Box S.4. Features of a Time to Correction Plot 12

Table SC.1. Vaccination schedule for doses in Colombia MOSV analysis 15

Table SN.1. Vaccination schedule for doses in Nigeria MOSV analysis 15

Map SC.1. Map of Colombia Showing Region Names 17

Table SN.2a. Visit-based MOSV analysis – Nigeria – BCG and OPV1-3 18

Table SN.2b. Visit-based MOSV analysis – Nigeria – DPT1-3 and MCV1 and any dose 19

Table SN.2c. Visit-based MOSV analysis – Nigeria – Any Dose, MOSVs per visit & Visits between MOSVs 20

Table SC.2a. Visit-based MOSV analysis – Colombia – BCG and OPV1-3 21

Table SC.2b. Visit-based MOSV analysis – Colombia – DPT1-3 and MCV1 22

Table SC.2c. Visit-based MOSV analysis – Colombia – Any dose, MOSVs per visit & Visits between MOSVs 23

Table SN.3a. Child-based MOSV analysis – Nigeria – National Results 25

Figure SN.3. Child-based MOSV analysis – Nigeria – Subnational Detail 26

Table SN.3b. Child-based MOSV analysis – Nigeria – Subnational Detail 27

Table SC.3a. Child-based MOSV analysis – Colombia – National Results 28

Figure SC.3. Child-based MOSV analysis – Colombia - Subnational Detail 29

Table SC.3b. Child-based MOSV analysis – Colombia - Subnational Detail 30

Table SN.4. MOSV Consequences – Nigeria – Potential Coverage Increase – National Results 31

Table SC.4. MOSV Consequences – Colombia – Potential Coverage Increase – National Results 32

Table SN.5a. MOSV Consequences – Nigeria – Time to Correction – National Results 33

Table SN.5b. MOSV Consequences – Nigeria – Time to Correction – Subnational Detail 34

Table SC.5a. MOSV Consequences – Colombia – Potential Coverage Increase – National Results 35

Table SC.5b. MOSV Consequences – Colombia – Potential Coverage Increase – Subnational Detail 36

INCIDENCE OF VACCINE PREVENTABLE DISEASES

Figure SVPD.1. Incidence rates for vaccine preventable diseases for Colombia & Nigeria, 1980-2020 [1]

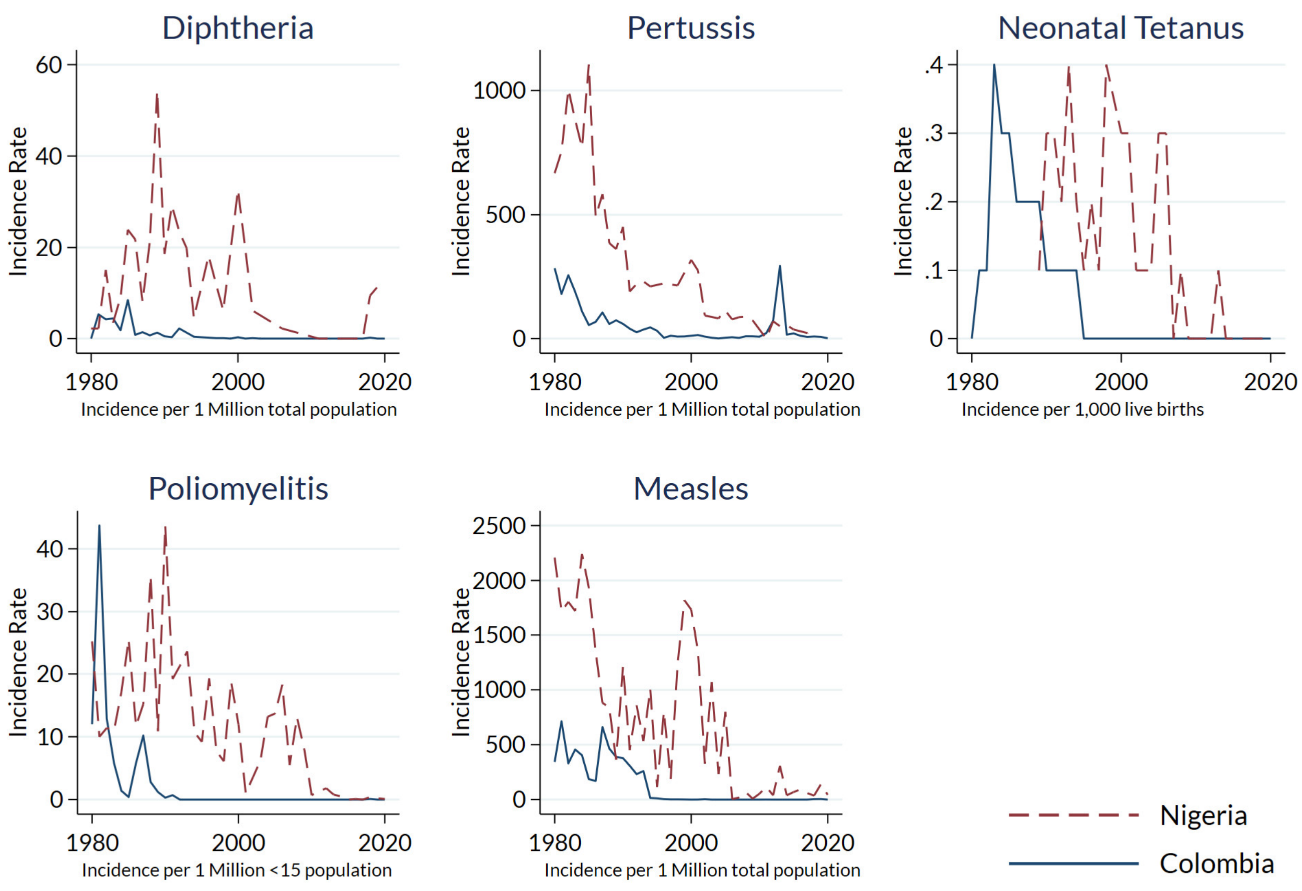

Source: WHO Immunization Data portal

PROVENANCE OF THE VCQI MOSV INDICATORS

MOV studies in the 1980s focused almost exclusively on a protocol for clinic-based visits. Caregivers with children are intercepted as they exit a health clinic and asked whether their child was vaccinated during the visit. Vaccination dates are copied from the child's home-based record (HBR or card) or from their facility-based record (FBR) and analyzed to determine whether they experienced MOVs at the clinic visit. Protocols for this type of study were developed at World Health Organization (WHO) headquarters in Geneva [2] and refined over the years at the Pan-American Health Organization (PAHO) [3,4] and again at WHO Geneva [5,6]. In clinic-based studies, even in recent years, the common analysis plan uses the child's vaccination dates from the card only to assess eligibility on a single day: the day of the clinic visit [7–9].

In the mid 1980s, the concept of analyzing whether all needed vaccines were administered during health service contacts was extended by one of us (FTC) to household surveys in Mozambique and subsequently Guinea Conakry – in the latter, caretakers were also asked about visits to health services for curative care [10]. In addition to estimating the prevalence of MOVs at preventive (Mozambique) and curative visits (Conakry and later also Central African Republic), survey authors estimated a "consequence indicator" of how much higher vaccination coverage would be if all the children in the study had received every eligible dose at every vaccination or growth monitoring visit and, in Conakry and CAR, at curative care [10,11]. Mothers' experience in being turned away from vaccination services (with reasons) and attitudes to vaccinating ill children were also assessed. While many of the early analyses were presumably conducted without using computers, later analyses of eligibility at every visit date used SAS programs written at the U.S. Centers for Disease Control. The programming was complex, as evidenced in archived source code from 1990/91 and documented with a comment early in the file:

```
|FUNCTION OF PROGRAM: DETERMINE MISSED OPPORTUNITIES FOR VACCINATION|
|                     DRIVE EVERYONE COMPLETELY NUTS                 |
```
[1]

The programming remains complex today. The VCQI modules that calculate MOV outcomes are some of the most complex of its 300+ Stata programs.

In the late 1980s, WHO sponsored development of DOS-based software named Coverage Survey Analysis System (COSAS)[2] and by 1991 COSAS documented and included indicators that assessed frequency of MOVs per clinic visit and per child, and consequence of MOVs at all of the child's documented vaccination dates [12,13]. COSAS was beloved by many immunization program staff[3] and reportedly used to analyze many surveys per year [14]. But the software was not upgraded over time and was rarely cited after the early 1990s. WHO's 2005 coverage survey reference manual and 2007 mid-level manager's training on coverage surveys give broad guidance for analyzing coverage survey data but do not describe analysis of MOSVs or any analysis using COSAS [15,16].

After COSAS there was an extended period from the mid-1990s to 2015 when WHO and PAHO had protocols for clinic based MOV studies, but there were no standardized guidance or tools for MOSV analyses using coverage survey data. This period corresponds precisely to that covered in the 2014 MOV literature review [17]. There were some interesting and reasonable analyses of MOVs using dates from coverage surveys and vaccination records review in that timeframe, but they did not employ standardized indicator definitions or analysis programs.

---

[1] From the author's (FTC) archive of project files.

[2] Personal communication with Eric Brenner, September 12, 2020.

[3] Personal communication with Pierre Claquin, Iqbal Hossain, Robert Steinglass & Alasdair Wylie.

In 2015 three of us (MLP & FTC & DAR) revisited definitions when we[4] authored the MOV sections of the working draft of WHO's updated vaccination coverage cluster survey reference manual (which was finalized in 2018) [18,19]. Note that the WHO manual uses the term MOV and in this manuscript we introduce the more specific phrase MOSV. That manual recommends using all the vaccination dates on each child's record to assess the frequency of MOSVs with (a) the child and (b) the health facility visit as denominators, as well as the consequence of MOSVs. In a companion effort, WHO commissioned a list of standard indicators that may be calculated with coverage survey data [20]. WHO selected a set of indicators to be developed more fully into software specifications and implemented in Stata programs. We call the list of indicators and the programs that calculate them, the Vaccination Coverage Quality Indicators (VCQI) [21].

GRAPHICAL EXAMINATION OF MISSED OPPORTUNITIES

Figure S.1 is useful to describe VCQI's indicator calculations. We use this type of figure to debug and validate VCQI indicator code, and these figures can be helpful for explaining and discussing MOVs with health workers. We formally call them "vaccination evidence and indicator plots" and informally "elbow plots". Text Box S.1 lists important features of the elbow plot. In principle, the plot shows the vaccination evidence from HBR and caregiver recall (and sometimes from a document such as a register at a health facility, if this is included in the household survey) and annotations to show the values of variables that are derived from the evidence. The annotations appear in the right margin and sometimes on the plot itself. There are many indicators in VCQI so to minimize clutter in this supplement, for each child we repeat the evidence across several plots and annotate each with only a subset of variables and indicators. Figure 1 is what we call a 'Crude MOV' plot – a phrase that is described more fully below. Text Box S.2 narrates the vaccination experience of the children in Figures S.1 through S.3a-b.

Figure S.1 shows a basic WHO Expanded Programme on Immunization (EPI) vaccine schedule with 8 vaccine-doses: BCG at birth; oral polio vaccine (OPV) and pentavalent vaccine (Penta) at 6, 10 and 14 weeks, and measles vaccine at 9 months.

We have developed a publicly-available R Shiny application for exploring child-level MOSV results in VCQI datasets, available at https://biostat-global-consulting.shinyapps.io/MOV_Tool_Public/. The application summarizes, for each dose in each stratum, the portions of children who received the dose at the first eligible opportunity, who had a corrected MOSV for the dose, and who had an uncorrected MOSV for the dose. The application also shows stratum-level summaries of the portion of children who experienced no MOSVs for any dose, and the portions who had MOSVs, all, some, or none of which were corrected by the time of the survey. For the subset of children who had corrected MOSVs, the delay between the initial missed opportunity and the visit when the dose was received is summarized in time to correction figures for each dose in each stratum.

Figures 3 and 4 in the manuscript were generated from the MOV Occurrence tab of the Shiny application, and Figures 6 and 7 in the manuscript were generated from the Time to Correction tab of the application. Figure S.4 and Text Box S.4 provide additional detail on interpreting the time to correction plots.

A series of videos orienting users to the R Shiny application is available on YouTube:

1. Introduction: https://youtu.be/GQ7Hcmh2czs
2. Exploring MOVs: https://youtu.be/oyyBJ-NzNug
3. Time to Correction: https://youtu.be/TS0ePSsbZRk
4. Data Requirements: https://youtu.be/gppzvsmKoVU

[4] Along with Dr. Pierre Claquin, WHO Consultant and Tony Burton, WHO Staff

Figure S.1. Vaccination Evidence and MOSV Indicators – Respondent 2 – had MOSVs for Penta2 and Penta3

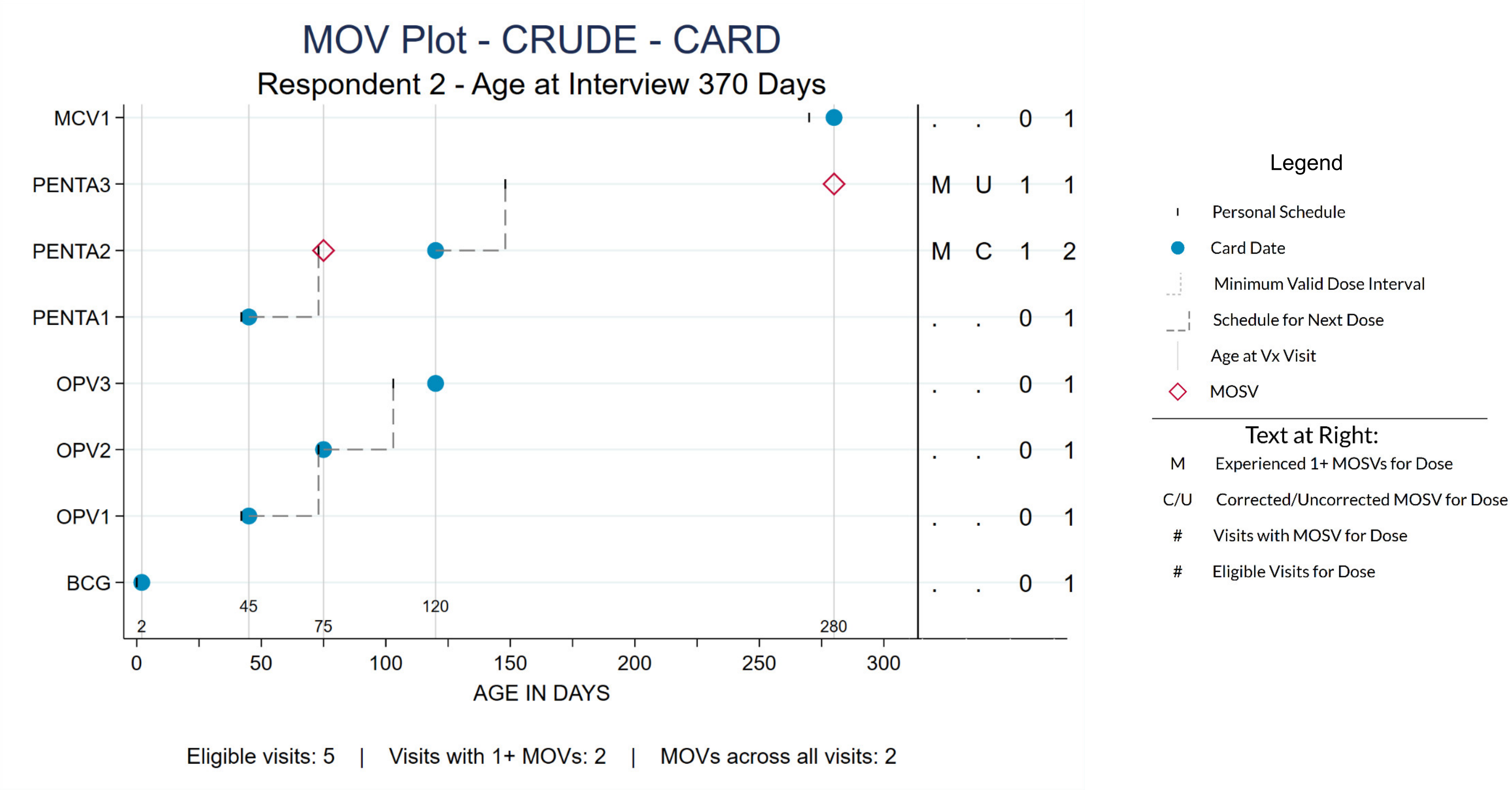

Text Box S.1. Features of an "Evidence and Indicators Plot"

- The child's age in days is shown on the x-axis and each vaccine-dose in the national schedule is represented with its own row.
- Doses that are scheduled to be given at birth appear at the bottom of the figure and those that are scheduled later appear near the top.
- Each series of multi-dose vaccines, like the 3-dose series of oral polio vaccine (OPV1-3) or pentavalent vaccine (Penta1-3) appear in adjacent rows.
- The age when the child becomes eligible to receive each dose is indicated with a small vertical bar (|) within the row.
- The age of eligibility for later doses in a series begin after a minimum interval has passed from the date when the earlier dose was received. These minimum intervals are indicated with right-angle connectors (*elbows*) that connect the date when the earlier dose was received to the date when the child becomes eligible for the next dose. These intra-dose minimum intervals are usually 4 weeks.
- Each health care contact visit is indicated with a tall vertical line that extends from the bottom to the top of the figure and lists the child's age in days near the bottom.
- For each vaccine-dose documented with a date on the HBR, the chart shows a colored dot at the intersection of the vertical visit line and the dose row.
- If the evidence of vaccination is from a tick mark on the HBR or from caregiver recall, the entire row is colored, because we do not know at what age the child received the dose.
- A child is eligible to receive the vaccine-dose at any age to the right of the vertical bar (|).
- If a health system visit occurs at an age when the child is eligible and if they do not receive the dose at that visit, we say that an MOSV has occurred, and we draw a red diamond around the intersection of the visit line and the dose row.
- In these figures, the text at the far right lists the values of several derived variables.
  - If the child experienced an MOSV for the dose, the first column holds the letter "M".
  - If the MOSV was later corrected, meaning that the child received the dose, but not at the first eligible visit, the second column holds the letter "C".
  - If the MOSV was uncorrected, i.e. the child had still not received the dose at the time the data were collected, the second column holds the letter "U".
  - The third column holds the number of contact visits at which the child experienced an MOSV for the dose.
  - The fourth column holds the number of contact visits when the child was eligible to receive the dose.

Text Box S.2. Vaccination experience for children in Figures S.1, S.2, and S.3a-b

The girl represented in Figure S.1 was 370 days old at the time of the survey.

- She received BCG at age 2 days.
- She received OPV1 and Penta1 at age 45 days meaning that she would be eligible for OPV2 and Penta2 from age 45+28 = 73 days.
- She received OPV2 but not Penta2 at age 75 days, so experienced an MOSV for Penta2 at that visit. She would be eligible for OPV3 at 75+28 = 103 days.
- She received OPV3 and Penta2 at age 120 days and would become eligible for Penta3 at 120+28 = 148 days.
- She received MCV at age 280 days, experiencing an MOSV for Penta3 at that visit.
- She was age 370 days at the time of the survey.
- This girl experienced two MOSVs, one for Penta2 and one for Penta3.
- The time to correction for the Penta2 MOSV was 120-75 = 45 days.
- At the time of the survey, the Penta3 MOSV was uncorrected, and she was 370-148 = 222 days overdue for that dose.
- If this child had not experienced any MOSVs, she would have been fully vaccinated at the age of 280 days.

The boy in Figure S.2 was 400 days old at the time of the survey.

- Received BCG, OPV-1-3, and MCV exactly on time.
- Did not receive Penta at all.
- Experienced MOSVs for Penta1 at age 42, 70, 98, and 270 days.
- The MOSVs are all uncorrected.
- If there had been no MOSVs, he would have also received Penta1-3 and been fully vaccinated at age 270 days.

The boy in Figures S.3a and S.3b was 388 days old at the time of the survey.

- He received BCG at birth and Penta1 at age 28 days, which is two weeks before the scheduled age of 42 days.
- He received OPV1 at 42 days and received all other doses exactly on schedule at 70, 98, and 270 days.
- Figure S.3a shows the crude dose analysis. The early dose of Penta is counted as dose 1 in the series and he is not credited with any MOSVs.
- Figure S.3b shows the valid dose analysis. The dose at 28 days is ignored because it was too early. On day 42, the child is credited with an MOSV for Penta at the time he received OPV1. That MOSV is corrected when the dose of Penta at day 70 is counted as dose 1, the dose of Penta on day 98 is counted as dose 2, and he is credited with an uncorrected Penta 3 MOSV at age 270 days when he receives only measles vaccine.

Figure S.2. Vaccination Evidence and MOSV Indicators - Respondent 3 – had MOSVs for Penta1

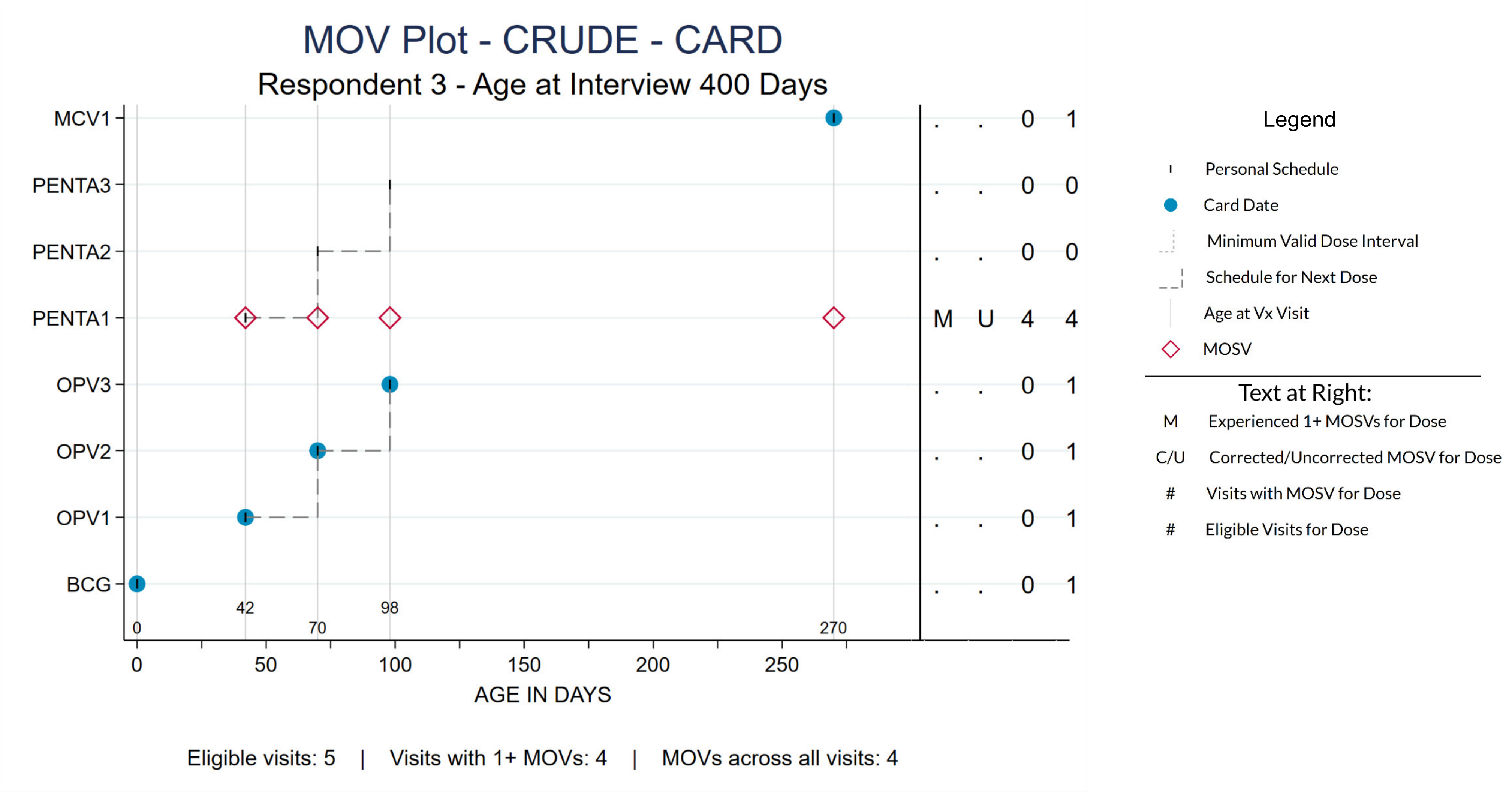

Figure S.3a. Vaccination Evidence and MOSV Indicators - Respondent 4 – VCQI crude dose analysis where early doses count

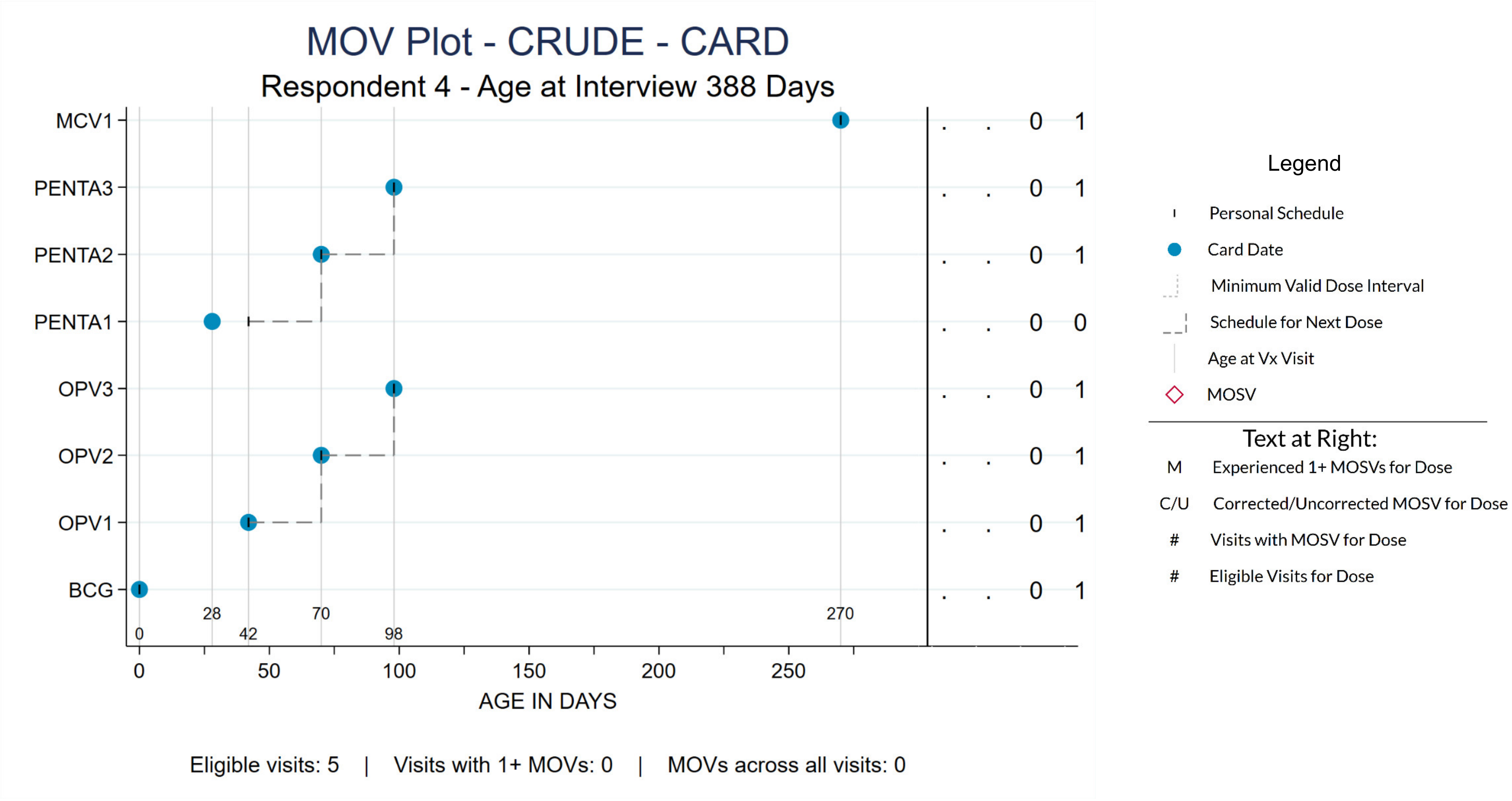

Figure S.3b. Vaccination Evidence and MOSV Indicators - Respondent 4 – VCQI valid dose analysis where early doses do not count

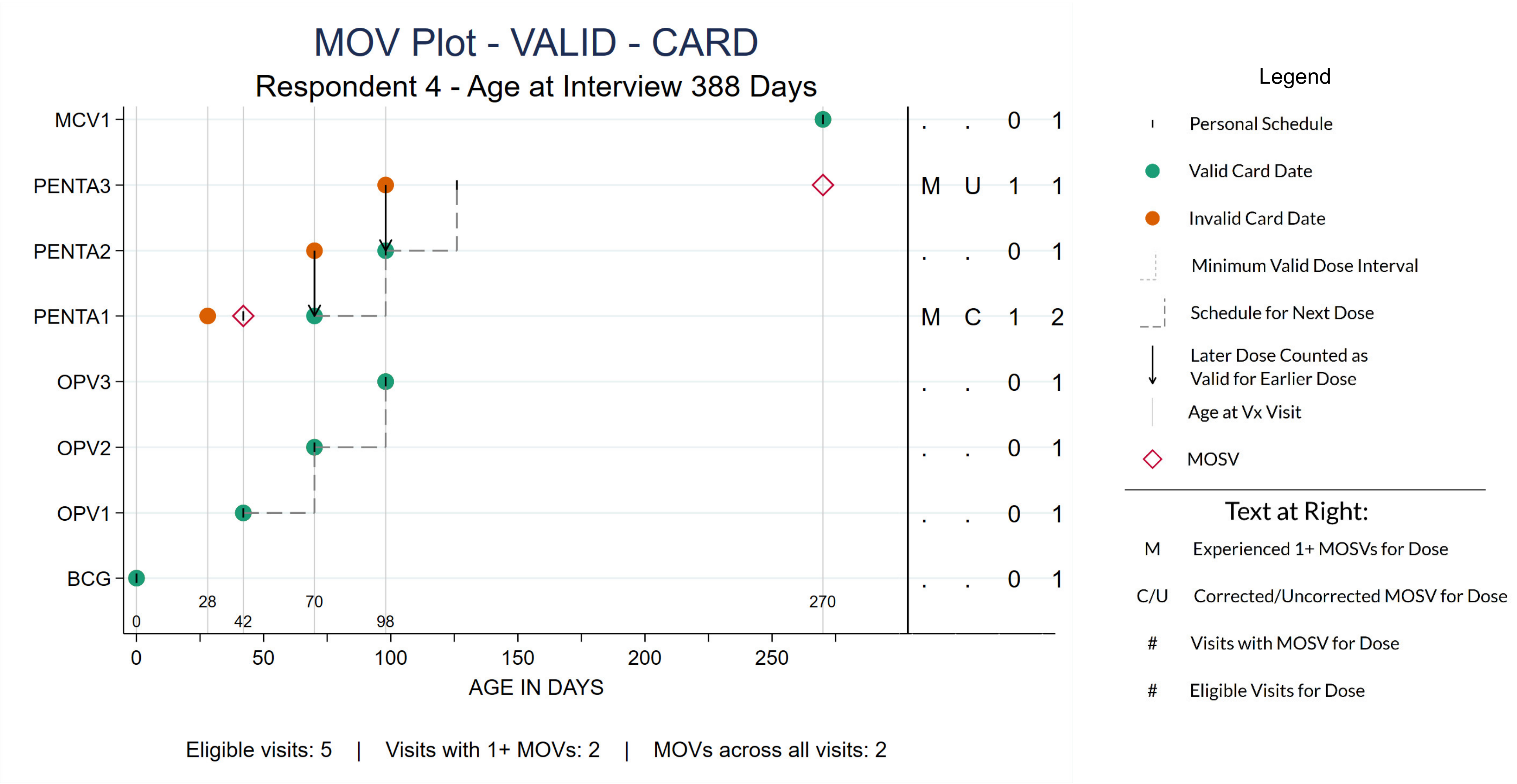

Figure S.4. Time to Correction Figure – Interpretation Guide

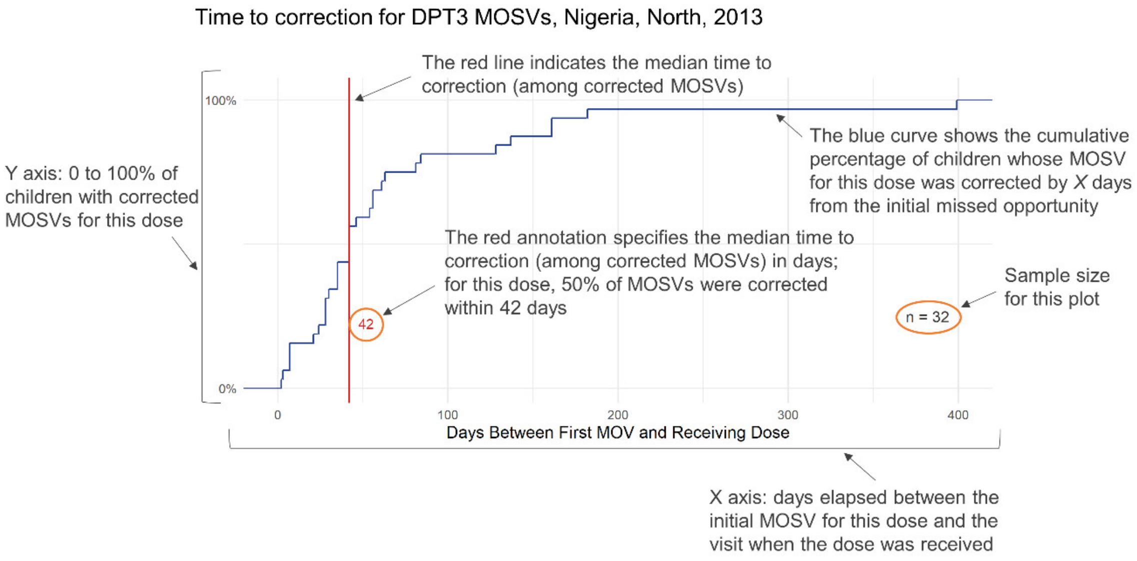

Text Box S.4. Features of a Time to Correction Plot

The time to correction figures in the manuscript show cumulative distributions of days until MOSV correction by stratum and dose. Figure S.4 is an annotated example of a single coverage curve figure, showing time to correction outcomes for children in a single stratum (the North zone of Nigeria) who had a corrected MOSV for a particular dose (DPT3). As time goes on, more children have their MOSVs corrected, until eventually 100% of the MOSVs that were corrected by the time of the survey have been corrected.

The blue curves in time to correction plots show the cumulative percentage of children whose MOSV was corrected by the number of days that have passed since the earliest opportunity to receive the dose. The red line and red annotation indicate the median time to correction in days. Sample size (n) is annotated in the bottom right of the plot.

MOSVs that were not corrected by the time of the survey are not represented in these plots because time to correction for those missed opportunities is not observed.

MOSV DATA QUALITY COMPLICATIONS

It is conceptually straightforward (though requiring complex programming) to identify MOSVs (or all MOVs if dates of other types of health contacts are available) and tally which were corrected, which were uncorrected, and to calculate the number of days the child spent under-protected because of their MOVs. But there are several practical issues that usually prevent the analyst from generalizing MOV results to the entire population.

Children without documented dates: In most household surveys in LMICs a notable portion of children do not show HBRs to interviewers so their MOVs cannot be assessed using the methods described here. This portion of children does not appear in the VCQI denominator for visit-based or child-based MOSV analyses and they never contribute to the numerator for children who would have received a valid dose if there had been no MOSVs.

Children with poor-quality documentation: There can be errors of omission and of commission for every element of the documented vaccination record: dates can be correct, incorrect, partial, illegible, missing, or written in the wrong spot on the HBR or FBR. Furthermore, the HBR can have different errors than the FBR, resulting in discordant evidence. Some doses that were not administered according to the documented evidence will be confidently vouched for by the caregiver (maybe she knows that she mistakenly left the HBR at home on the day of the most recent clinic visit).

Date data quality problems will have various consequences for MOV indicators. If evidence is from an unusable date or tick mark or from caregiver recall, then the dose cannot be assessed for MOVs. The analyst (and software) have no way of knowing at what visit or age the child received that dose. They also have no way of knowing whether the child experienced MOVs at the visit when they received the date-deficient dose. This removes some doses or entire visits from indicator denominators. If the date is complete but wrong, it can look like a legitimate visit that never occurred and it can add spurious observations to the denominator and the numerators. The errant dates appear in the analysis as visit dates and the software mistakenly counts some MOVs that never happened. Some errant dates will contribute to numerators and denominators (if the child would have been eligible for doses on the incorrect date) and some will be more benign if they fall in a time period when the child would not be eligible for any doses.

MOSV CALCULATION COMPLICATIONS

VCQI is flexible and can accommodate differences in country vaccination schedule, type of survey sampling, whether the analysis should use survey weights and different combinations of vaccination evidence. No matter which combination is appropriate, VCQI analyzes the MOVs using the same programs, which are responsive to different input parameters.

That flexibility comes at the cost of the software being complex. Although VCQI is comprised of more than 300 Stata programs (.ado files), the user only interacts with one program, called the *control program* where they specify parameters to describe the vaccination schedule, the survey parameters, and which indicators to calculate. The vaccination schedule is specified with up to three parameters per dose: minimum age to receive a valid dose; optionally, a maximum age when the dose is valid; and for doses in a series, the minimum intra-dose interval.

There are two aspects of MOV calculation that require special flexibility.

Long intra-dose schedule versus minimum interval for valid doses: In countries where childhood diseases are prevalent, vaccination visits are usually scheduled four weeks apart so children can reach full protection as early as possible. In countries where disease is less prevalent, the vaccination visits

are sometimes spaced out farther. In Latin American countries, the OPV/Penta1-3 visits are scheduled eight weeks apart. A later dose is valid any time after four weeks have lapsed but the child is not scheduled to come back for eight weeks. This presents a computational challenge in evaluating eligibility. VCQI addresses this challenge using two parameters: the scheduled age for each dose, and the minimum interval required for the later dose to be considered valid.

Doses administered too early: Consider the child whose history is portrayed in Figure S.3a. The card says they received Penta1 at age 28 days when it was scheduled at 42 days. They receive Penta2 and 3 on time at 70 and 98 days. Dose 1 was invalid. What should the healthcare worker do when the child returns for MCV at age 270 days? Administer a fourth dose of Penta, which would be the child's third *valid* dose? If yes, where would they write that date on the HBR? The spaces for Penta1, 2 and 3 are already filled. What are the chances that the worker will even notice that the dose was early? And now to the point for MOVs, should VCQI count an MOV if the worker fails to administer the third valid dose along with MCV at age 270 days?

VCQI calculates MOSV outcomes two ways and then tabulates and graphs the set of outcomes that the user requests. The so-called *crude analysis* counts early doses and valid doses alike. If there is evidence on the HBR that they received the dose, then the clinic worker is not expected to repeat invalid doses at later visits, and VCQI does not count an MOSV if they fail to do so. Figure S.3a shows results of the crude analysis; VCQI did not count an MOSV for Penta3 at 270 days. In the more strict, so-called *valid analysis*, early doses do not count, and VCQI counts an MOSV if the child returns later but does not receive the full complement of valid doses. Figure S.3b shows results of the valid analysis for the same child, where VCQI finds an MOSV for Penta3. The valid analysis yields more MOSVs than the crude analysis. The crude analysis probably corresponds to the practice in many countries: If the dose appears on the card, it is not repeated – even if it was early. The valid analysis is more concerned with maximizing the number of valid doses and thereby maximizing the likelihood the child will develop immunity. When we run VCQI, it is our practice to examine the crude outcome first. If the crude MOSV numbers are concerning, there is no need to consult the valid analysis. If the crude analysis is not concerning, then it may be worthwhile to also examine results of the valid analysis.

In our opinion, the topic of crude versus valid analysis warrants more exploration. It would be helpful to have clear guidance from WHO and for countries to turn extant guidance [22,23] into clear national policies concerning whether health workers should repeat doses that were given too early, and if so, under what circumstances. In the early 2010s many countries only gave first-year-of-life (1YL) doses to children who were still in the first year of life. In recent years, some second year of life (2YL) doses have been introduced into the schedule and countries are encouraged to catch-up for doses that were missed in infancy. It would be feasible to recommend catching up for doses given too early, but there are practical concerns, such as how those repeated doses would be recorded on the HBR. At this time in LMICs the appropriate first MOSV analysis is the more conservative crude analysis because it is likely consistent with their vaccination practices and because if it shows a notable prevalence of MOSVs then stakeholder engagement is warranted for reducing MOSVs. If the crude analysis outcomes are acceptably low then one might look at the valid analysis, but the results will only differ if there are a lot of early doses, which should already have been observed when looking at other timeliness indicators that we have not detailed here.

VACCINATION SCHEDULES FOR MOSV ANALYSES

Tables SC.1 and SN.1 list the ages at which the MOSV analysis doses were scheduled in Colombia and Nigeria for the years of the surveys. Other doses in the datasets were scheduled for these same ages, but only the schedules of the analysis doses are relevant for the MOSV results.

Table SC.1. Vaccination schedule for doses in Colombia MOSV analysis

Age	Doses Analyzed for MOSVs
Birth	BCG
2 months	OPV1, DPT1
4 months	OPV2, DPT2
6 months	OPV3 DPT3
1 year	MCV

Table SN.1. Vaccination schedule for doses in Nigeria MOSV analysis

Age	Doses Analyzed for MOSVs
Birth	BCG
6 weeks	OPV1, DPT1
10 weeks	OPV2, DPT2
14 weeks	OPV3 DPT3
9 months	MCV

REFERENCES

1. World Health Organization WHO Vaccine Preventable Disease Incidence Data. Available online: https://whowiise.blob.core.windows.net/upload/incidence-rate.xlsx (accessed on 5 July 2021).
2. WHO EPI Study of Immunization Status and Reasons for Postponing Immunizations: Protocol 1984.
3. Pan American Health Organization Methodology for the Evaluation of Missed Opportunities for Vaccination 2013.
4. Rosenbaum, J. Revised Protocol for the Assessment of Missed Opportunities for Vaccination 1988.
5. Sato, P. Protocol for the Assessment of Missed Opportunities for Immunization 1988.
6. World Health Organization *Methodology for the Assessment of Missed Opportunities for Vaccination*; World Health Organization: Geneva, Switzerland, 2017; ISBN 978-92-4-151295-4.
7. Li, A.J.; Tabu, C.; Shendale, S.; Sergon, K.; Okoth, P.O.; Mugoya, I.K.; Machekanyanga, Z.; Onuekwusi, I.U.; Sanderson, C.; Ogbuanu, I.U. Assessment of Missed Opportunities for Vaccination in Kenyan Health Facilities, 2016. *PLOS ONE* 2020, 17.
8. Li, A.J.; Peiris, T.S.R.; Sanderson, C.; Nic Lochlainn, L.; Mausiry, M.; da Silva, R.B.J.B.M.; Ogbuanu, I.U. Opportunities to Improve Vaccination Coverage in a Country with a Fledgling Health System: Findings from an Assessment of Missed Opportunities for Vaccination among Health Center Attendees—Timor Leste, 2016. *Vaccine* 2019, *37*, 4281–4290, doi:10.1016/j.vaccine.2019.06.041.
9. Ogbuanu, I.U.; Li, A.J.; Anya, B.M.; Tamadji, M.; Chirwa, G.; Chiwaya, K.W.; Djalal, M.E.-H.; Cheikh, D.; Machekanyanga, Z.; Okeibunor, J.; et al. Can Vaccination Coverage Be Improved by Reducing Missed Opportunities for Vaccination? Findings from Assessments in Chad and Malawi Using the New WHO Methodology. *PLoS ONE* 2019, *14*, e0210648, doi:10.1371/journal.pone.0210648.
10. Cutts, F.T.; Zell, E.; Soares, A.C.; Diallo, S. Obstacles to Achieving Immunization for All 2000: Missed Immunization Opportunities and Inappropriately Timed Immunization. *Journal of Tropical Pediatrics* 1991, *37*, 153–158.
11. Kahn, J.G.; Mokdad, A.H.; Deming, M.S. Avoiding Missed Opportunities for Immunization in the Central African Republic: Potential Impact on Vaccination Coverage. 1995, *73*, 9.
12. Desvé, G.; Havreng, J.-F.; Brenner, E. Coverage Survey Analysis System (COSAS) 4.3 - Software for EPI 30 Cluster Coverage Surveys 1991.
13. Grabowsky, M. *Missed Opportunities for Immunization*; Resources for Child Health Project: Arlington, VA, 1991.
14. Hutchins, S.S.; Jansen, H.A.F.M.; Robertson, S.E. Missed Opportunities for Immunization - Review of Studies from Developing and Industrialized Countries 1992.
15. WHO Training for Mid-Level Managers: The EPI Coverage Survey 2007.
16. World Health Organization *Immunization Coverage Cluster Survey: Reference Manual*; World Health Organization: Geneva, Switzerland, 2005.
17. Sridhar, S.; Maleq, N.; Guillermet, E.; Colombini, A.; Gessner, B.D. A Systematic Literature Review of Missed Opportunities for Immunization in Low- and Middle-Income Countries. *Vaccine* 2014, *32*, 6870–6879, doi:10.1016/j.vaccine.2014.10.063.
18. World Health Organization. Vaccination Coverage Cluster Surveys: Reference Manual; (No. WHO/IVB/18.09). World Health Organization: Geneva, Switzerland, 2018. https://apps.who.int/iris/handle/10665/272820. License: CC BY-NC-SA 3.0 IGO
19. World Health Organization Vaccination Coverage Cluster Surveys: Reference Manual - Working Draft 2015.
20. Rhoda, D.A. *VCQI Indicator List with Specifications, Draft Version 2.2*; Biostat Global Consulting, 2021.
21. Biostat Global Consulting Vaccination Coverage Quality Indicators (VCQI). Resources available online: http://www.biostatglobal.com/VCQI_resources.html.
22. World Health Organization Summary of WHO Position Papers - Recommendations for Routine Immunization. Available online: http://www.who.int/immunization/policy/Immunization_routine_table1.pdf (accessed on 31 May 2021).
23. World Health Organization Summary of WHO Position Papers - Recommended Routine Immunizations for Children. Available online: http://www.who.int/immunization/policy/Immunization_routine_table2.pdf (accessed on 31 May 2021).

ADDITIONAL DETAILS TO SUPPLEMENT FIGURES IN THE MANUSCRIPT

Map SC.1. Map of Colombia Showing Region Names

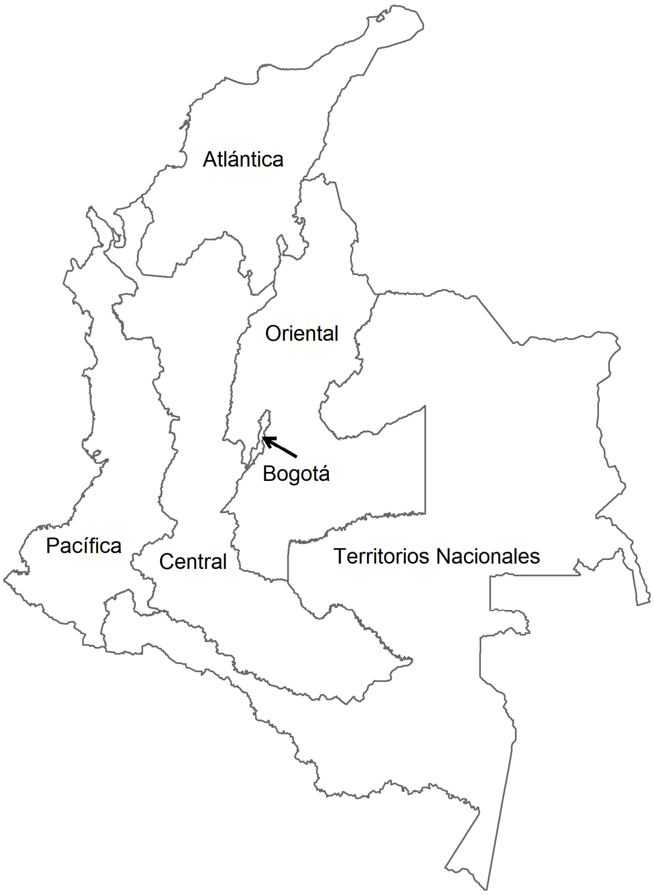

Table SN.2a. Visit-based MOSV analysis – Nigeria – BCG and OPV1-3

Year	Stratum	Visits with MOSV for BCG (%)	N	Visits with MOSV for OPV1 (%)	N	Visits with MOSV for OPV2 (%)	N	Visits with MOSV for OPV3 (%)	N
1990	Nigeria	5.4	441	16.0	438	1.0	300	3.6	253
1999	Nigeria	16.5	236	47.4	268	5.9	135	22.2	117
2003	Nigeria	13.5	244	46.4	233	7.1	112	18.6	97
2008	Nigeria	35.3	1,548	37.7	1,219	12.9	774	10.0	647
2013	Nigeria	26.7	2,068	34.7	1,750	12.0	1,237	12.6	1,102
2018	Nigeria	24.2	2,774	13.9	1,668	4.1	1,403	4.7	1,373
1990	*North*	8.7	172	13.1	153	3.2	93	5.6	71
1999	*North*	31.7	41	51.3	39	.0	18	38.5	13
2003	*North*	12.6	127	56.3	135	9.1	44	23.7	38
2008	*North*	49.6	714	44.3	503	23.5	302	16.1	230
2013	*North*	33.8	904	38.5	736	12.4	469	17.1	391
2018	*North*	27.9	1,644	17.8	987	6.3	766	5.1	707
1990	*South*	3.3	269	17.5	285	.0	207	2.7	182
1999	*South*	14.1	142	46.2	173	8.8	91	25.3	79
2003	*South*	14.5	117	32.7	98	5.9	68	15.3	59
2008	*South*	23.1	834	33.0	716	6.1	472	6.7	417
2013	*South*	21.1	1,164	32.1	1,014	11.8	768	10.1	711
2018	*South*	18.8	1,130	8.2	681	1.4	637	4.4	666
Scale		100.0		100.0		100.0		100.0	

Notes for this table appear after Table SN.2c

Table SN.2b. Visit-based MOSV analysis – Nigeria – DPT1-3 and MCV1 and any dose

Year	Stratum	Visits with MOSV for DPT1 (%)	N	Visits with MOSV for DPT2 (%)	N	Visits with MOSV for DPT3 (%)	N	Visits with MOSV for MCV1 (%)	N	Visits with MOSV for any dose (%)	N
1990	Nigeria	17.7	440	1.0	301	2.8	250	25.6	324	14.1	1,351
1999	Nigeria	50.5	277	7.8	129	16.2	105	28.2	149	38.5	732
2003	Nigeria	42.0	238	5.8	120	15.4	104	32.3	124	33.3	655
2008	Nigeria	29.2	1,267	6.6	828	6.8	766	27.0	937	32.7	4,222
2013	Nigeria	36.2	1,963	14.1	1,332	12.6	1,164	24.3	1,253	29.8	6,599
2018	Nigeria	15.4	1,711	3.1	1,424	3.9	1,384	23.3	1,350	16.9	7,690
1990	*North*	17.5	154	1.1	91	4.3	70	33.1	139	21.0	424
1999	*North*	25.7	35	8.0	25	40.0	20	48.4	31	46.8	111
2003	*North*	42.2	128	7.5	53	16.7	48	44.8	67	41.8	318
2008	*North*	34.6	534	10.7	317	8.8	284	31.3	386	45.2	1,650
2013	*North*	36.1	795	15.7	508	16.2	421	31.9	518	35.6	2,528
2018	*North*	19.8	1,019	4.5	771	5.2	718	32.2	733	22.0	4,155
1990	*South*	17.8	286	1.0	210	2.2	180	20.0	185	10.9	927
1999	*South*	54.5	178	7.6	79	14.3	63	20.9	91	37.5	472
2003	*South*	41.8	110	4.5	67	14.3	56	17.5	57	25.2	337
2008	*South*	25.2	733	4.1	511	5.6	482	24.0	551	24.7	2,572
2013	*South*	36.3	1,168	13.1	824	10.6	743	19.0	735	26.2	4,071
2018	*South*	9.0	692	1.4	653	2.6	666	12.8	617	10.9	3,535
Scale		100.0		100.0		100.0		100.0		100.0	

Notes for this table appear after Table SN.2c

Table SN.2c. Visit-based MOSV analysis – Nigeria – Any Dose, MOSVs per visit & Visits between MOSVs

Year	Stratum	MOSVs per Visit	N	Visits between MOSVs
1990	Nigeria	0.205	1,351	4.9
1999	Nigeria	0.559	732	1.8
2003	Nigeria	0.504	655	2.0
2008	Nigeria	0.450	4,222	2.2
2013	Nigeria	0.424	6,599	2.4
2018	Nigeria	0.221	7,690	4.5
1990	*North*	0.281	424	3.6
1999	*North*	0.649	111	1.5
2003	*North*	0.632	318	1.6
2008	*North*	0.636	1,650	1.6
2013	*North*	0.520	2,528	1.9
2018	*North*	0.296	4,155	3.4
1990	*South*	0.170	927	5.9
1999	*South*	0.549	472	1.8
2003	*South*	0.383	337	2.6
2008	*South*	0.331	2,572	3.0
2013	*South*	0.365	4,071	2.7
2018	*South*	0.134	3,535	7.5
Scale		1.000		10

Notes
Percent of visits where children were eligible for the dose and did not receive it.
This is a crude dose analysis, meaning that early doses are accepted and counted in this analysis.
The final two measures on this sheet, MOSVs per visit and Visits between MOSVs are NOT percentages.
These analyses are unweighted.
The 1999 survey included a stratum named 'Central' which is included in the rows labeled 'Nigeria' but is excluded from the North vs. South comparison.
Blue columns labeled (%) are shaded such that 100% would fill the entire table cell.
The orange column labeled 'MOSVs per visit' are shaded such that 1.0 would fill the entire cell.
The pink column labeled 'Visits between MOSVs' is shaded such that 10.0 would fill the entire table cell. It can be interpreted as showing e.g. that an MOSV occurred on average once in every 3 visits for the South in 2008, compared to once in every 8 visits in the South in 2018.

Table SC.2a. Visit-based MOSV analysis – Colombia – BCG and OPV1-3

Year	Stratum	Visits with MOSV for BCG (%)	N	Visits with MOSV for OPV1 (%)	N	Visits with MOSV for OPV2 (%)	N	Visits with MOSV for OPV3 (%)	N
1995	Colombia	29.5	786	7.9	534	6.3	378	7.8	322
2000		43.4	1,104	20.6	762	18.3	613	22.5	564
2005		20.9	2,628	28.7	2,617	5.3	1,657	8.3	1,518
2010		10.3	2,957	10.5	2,782	2.1	2,482	4.4	2,356
1995	Atlántica	42.4	243	14.4	153	12.1	91	10.8	65
2000		59.2	331	25.1	171	28.1	121	28.4	102
2005		29.7	817	34.0	803	6.4	452	12.6	405
2010		17.3	773	6.2	662	2.9	611	5.3	568
1995	Bogotá	15.0	80	11.1	63	7.4	54	9.3	43
2000		17.5	114	17.0	106	15.2	99	10.0	80
2005		4.7	127	41.5	164	4.2	96	4.2	95
2010		.8	122	28.9	152	.9	112	2.8	109
1995	Central	21.1	190	5.1	137	1.9	105	10.0	100
2000		34.0	288	24.2	244	15.7	178	22.4	170
2005		19.4	572	28.6	566	5.2	348	7.8	320
2010		6.1	575	9.7	555	1.6	513	3.3	511
1995	Oriental	21.1	128	3.2	95	6.7	60	.0	52
2000		46.5	202	12.3	122	18.3	120	24.4	123
2005		11.3	345	32.8	381	3.7	244	8.2	232
2010		6.5	397	15.4	408	1.5	323	7.9	318
1995	Pacífica	34.5	145	3.5	86	4.4	68	6.5	62
2000		42.0	169	18.5	119	13.7	95	24.7	89
2005		21.4	406	23.2	383	5.9	273	7.3	247
2010		12.1	429	7.7	378	2.3	349	3.6	333
2005	Territorios Nacionales	17.7	361	10.6	320	4.9	244	4.1	219
2010		8.8	661	9.6	627	2.3	574	3.3	517
Scale		100.0		100.0		100.0		100.0	

Notes for this table appear after Table SC.2c

Table SC.2b. Visit-based MOSV analysis – Colombia – DPT1-3 and MCV1

Year	Stratum	Visits with MOSV for DPT1 (%)	N	Visits with MOSV for DPT2 (%)	N	Visits with MOSV for DPT3 (%)	N	Visits with MOSV for MCV (%)	N
1995	Colombia	8.4	536	4.4	361	7.5	319	22.5	71
2000		22.4	780	20.3	629	21.9	584	22.9	516
2005		6.7	2,137	4.2	1,756	8.8	1,661	9.8	1,575
2010		5.2	2,623	1.8	2,482	3.5	2,344	7.1	2,228
1995	Atlántica	10.9	147	6.3	80	16.4	67	17.9	28
2000		23.1	173	26.4	121	29.4	109	24.0	104
2005		8.0	624	5.4	481	10.1	436	14.6	458
2010		6.0	650	2.2	601	4.5	559	7.8	540
1995	Bogotá	13.6	66	7.5	53	7.0	43	12.5	8
2000		20.7	111	13.3	98	11.6	86	14.3	70
2005		9.2	120	4.5	112	3.6	110	5.1	78
2010		2.5	122	.0	122	2.6	117	3.8	106
1995	Central	5.1	136	1.0	102	5.3	94	53.8	13
2000		28.3	251	21.9	192	21.6	171	25.3	158
2005		4.5	448	4.3	374	7.8	346	7.0	341
2010		9.2	544	1.4	507	2.6	501	6.1	490
1995	Oriental	5.2	96	6.7	60	.0	52	16.7	12
2000		15.1	126	16.9	118	26.4	129	25.5	110
2005		4.0	300	1.9	263	10.5	277	8.8	227
2010		2.3	352	3.3	331	3.3	307	4.0	301
1995	Pacífica	8.8	91	3.0	66	7.9	63	10.0	10
2000		18.5	119	21.0	100	16.9	89	20.3	74
2005		10.9	341	4.3	281	8.5	258	8.9	224
2010		3.3	361	1.7	354	5.0	341	8.3	300
2005	Territorios Nacionales	4.3	304	4.1	245	8.5	234	8.1	247
2010		4.2	594	1.4	567	2.5	519	9.2	491
Scale		100.0		100.0		100.0		100.0	

Notes for this table appear after Table SC.2c

Table SC.2c. Visit-based MOSV analysis – Colombia – Any dose, MOSVs per visit & Visits between MOSVs

Year	Stratum	Visits with MOSV for any dose (%)	N	MOSVs per Visit	N	Visits between MOSVs
1995	Colombia	18.5	1,842	0.230	1,842	4.3
2000		33.1	3,301	0.431	3,301	2.3
2005		18.7	9,368	0.217	9,368	4.6
2010		8.5	12,391	0.095	12,391	10.5
1995	Atlántica	29.1	484	0.372	484	2.7
2000		45.4	764	0.564	764	1.8
2005		24.9	2,627	0.298	2,627	3.4
2010		9.8	3,015	0.113	3,015	8.8
1995	Bogotá	14.3	230	0.191	230	5.2
2000		18.0	450	0.260	450	3.8
2005		17.0	546	0.194	546	5.2
2010		9.2	588	0.100	588	10.0
1995	Central	13.1	495	0.160	495	6.3
2000		31.3	973	0.424	973	2.4
2005		18.2	2,019	0.200	2,019	5.0
2010		7.5	2,589	0.083	2,589	12.1
1995	Oriental	11.8	321	0.140	321	7.1
2000		32.0	606	0.432	606	2.3
2005		16.5	1,386	0.186	1,386	5.4
2010		8.8	1,713	0.093	1,713	10.7
1995	Pacífica	20.5	312	0.244	312	4.1
2000		32.5	508	0.396	508	2.5
2005		17.8	1,433	0.210	1,433	4.8
2010		8.4	1,744	0.092	1,744	10.8
2005	Territorios Nacionales	11.4	1,357	0.134	1,357	7.5
2010		7.7	2,742	0.087	2,742	11.5
Scale		100.0		1.000		15.0

Notes:
Percent of visits where children were eligible for the dose and did not receive it.
This is a crude dose analysis, meaning that early doses are accepted and counted in this analysis.
The final two measures on this sheet, MOSVs per visit and Visits between MOSVs are NOT percentages.
These analyses are unweighted.
The stratum named 'Territorios Nacionales' appears only in the 2005 and 2010 surveys.
Blue columns labeled (%) are shaded such that 100% would fill the entire table cell.
The orange column labeled 'MOSVs per visit' are shaded such that 1.0 would fill the entire cell.
The pink column labeled 'Visits between MOSVs' is shaded such that 15.0 would fill the entire table cell.

Table SN.3a. Child-based MOSV analysis – Nigeria – National Results

Stratum	BCG	DPT1	DPT2	DPT3	MCV1	OPV1	OPV2	OPV3	Summary
Nigeria 1990	408 | 9 | 6	299 | 63 | 15	295 | 3 | 0	242 | 1 | 6	218 | 23 | 40	309 | 59 | 10	294 | 3 | 0	241 | 3 | 6	294 | 82 | 5 | 57
Nigeria 1999	184 | 13 | 7	82 | 55 | 27	115 | 4 | 6	86 | 2 | 14	96 | 11 | 14	65 | 76 | 15	122 | 5 | 2	90 | 1 | 22	75 | 57 | 38 | 40
Nigeria 2003	201 | 10 | 10	94 | 44 | 28	110 | 3 | 4	82 | 6 | 9	74 | 10 | 21	76 | 47 | 31	102 | 2 | 6	76 | 3 | 12	97 | 49 | 27 | 51
Nigeria 2008	825 | 176 | 126	624 | 271 | 50	736 | 37 | 12	684 | 30 | 15	593 | 91 | 91	486 | 272 | 79	638 | 36 | 33	554 | 28 | 26	506 | 422 | 125 | 188
Nigeria 2013	1256 | 260 | 66	762 | 490 | 46	1023 | 121 | 19	927 | 90 | 27	838 | 110 | 107	700 | 442 | 43	994 | 94 | 18	879 | 84 | 27	732 | 721 | 121 | 142
Nigeria 2018	1844 | 259 | 130	1288 | 159 | 67	1346 | 34 | 10	1301 | 29 | 16	960 | 75 | 128	1288 | 148 | 49	1317 | 29 | 19	1277 | 31 | 22	1514 | 439 | 71 | 242

Calculations use crude measures of MOV. Dose columns show counts: Vaccinated at First Eligible Opportunity | MOV - Later Corrected | MOV - Uncorrected.

Summary column shows, for each stratum, counts: No MOVs | All MOVs Corrected | Mixture Corrected/Uncorrected | All MOVs Uncorrected.
Summary column calculated using the following doses: BCG, DPT1, DPT2, DPT3, MCV1, OPV1, OPV2, OPV3

Figure SN.3. Child-based MOSV analysis – Nigeria – Subnational Detail

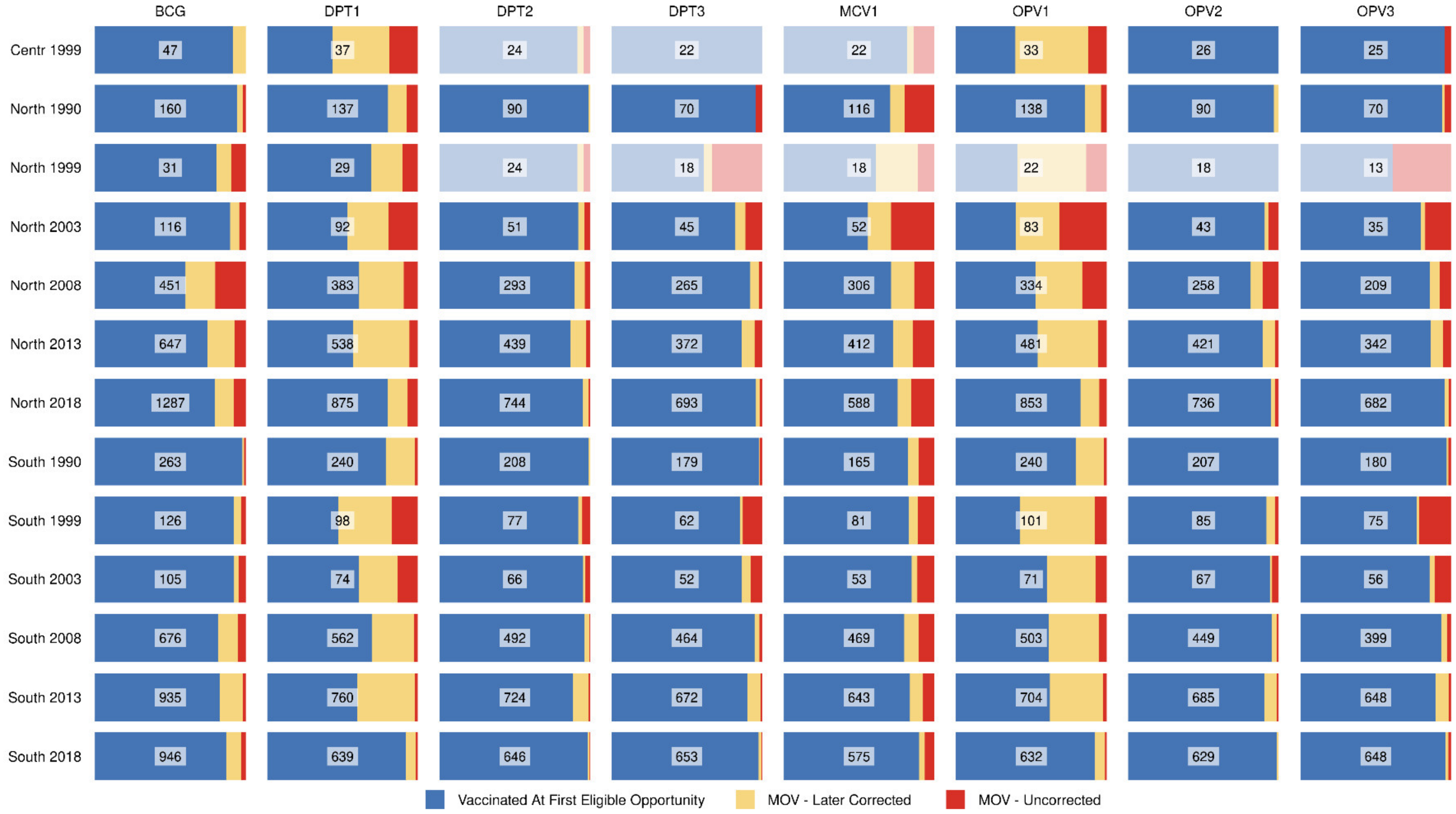

	NM	AC	SC	NC
Centr 1999	38.3	29.8	14.9	17.0
North 1990	61.4	17.5	1.2	19.9
North 1999	30.3	21.2	21.2	27.3
North 2003	33.9	22.9	15.3	28.0
North 2008	30.3	32.2	14.2	23.2
North 2013	40.6	36.8	10.1	12.5
North 2018	60.1	22.3	3.8	13.8
South 1990	70.6	19.5	1.1	8.8
South 1999	36.2	27.7	18.5	17.7
South 2003	53.8	20.8	8.5	17.0
South 2008	48.3	35.3	7.1	9.3
South 2013	44.1	45.9	4.8	5.2
South 2018	76.0	15.4	2.2	6.5

Crude measures of MOV shown. Figure shows suppressed output for bars where n < 25. Sample size is shown on each bar.

The stratum summary table shows information for the following doses: BCG, DPT1, DPT2, DPT3, MCV1, OPV1, OPV2, OPV3.

MOV Summary by Stratum. % kids with:
NM = no MOVs; AC = all MOVs corrected;
SC = some corrected; NC = none corrected.
Percentages in each row sum to 100%.

Table SN.3b. Child-based MOSV analysis – Nigeria – Subnational Detail

Stratum	BCG	DPT1	DPT2	DPT3	MCV1	OPV1	OPV2	OPV3	Summary
Centr 1999	43 | 4 | 0	16 | 14 | 7	22 | 1 | 1	22 | 0 | 0	18 | 1 | 3	13 | 16 | 4	26 | 0 | 0	24 | 0 | 1	18 | 14 | 7 | 8
North 1990	151 | 6 | 3	110 | 17 | 10	89 | 1 | 0	67 | 0 | 3	82 | 11 | 23	118 | 15 | 5	87 | 3 | 0	66 | 1 | 3	102 | 29 | 2 | 33
North 1999	25 | 3 | 3	20 | 6 | 3	22 | 1 | 1	11 | 1 | 6	11 | 5 | 2	9 | 10 | 3	18 | 0 | 0	8 | 0 | 5	10 | 7 | 7 | 9
North 2003	104 | 7 | 5	49 | 25 | 18	47 | 2 | 2	37 | 3 | 5	29 | 8 | 15	33 | 24 | 26	39 | 1 | 3	28 | 1 | 6	40 | 27 | 18 | 33
North 2008	271 | 89 | 91	234 | 113 | 36	263 | 20 | 10	244 | 15 | 6	218 | 47 | 41	176 | 104 | 54	210 | 21 | 27	180 | 13 | 16	158 | 168 | 74 | 121
North 2013	482 | 116 | 49	307 | 201 | 30	381 | 47 | 11	321 | 32 | 19	299 | 54 | 59	261 | 192 | 28	376 | 35 | 10	296 | 28 | 18	295 | 267 | 73 | 91
North 2018	1022 | 164 | 101	699 | 118 | 58	709 | 27 | 8	664 | 17 | 12	444 | 53 | 91	705 | 106 | 42	697 | 21 | 18	653 | 18 | 11	784 | 291 | 50 | 180
South 1990	257 | 3 | 3	189 | 46 | 5	206 | 2 | 0	175 | 1 | 3	136 | 12 | 17	191 | 44 | 5	207 | 0 | 0	175 | 2 | 3	192 | 53 | 3 | 24
South 1999	116 | 6 | 4	46 | 35 | 17	71 | 2 | 4	53 | 1 | 8	67 | 5 | 9	43 | 50 | 8	78 | 5 | 2	58 | 1 | 16	47 | 36 | 24 | 23
South 2003	97 | 3 | 5	45 | 19 | 10	63 | 1 | 2	45 | 3 | 4	45 | 2 | 6	43 | 23 | 5	63 | 1 | 3	48 | 2 | 6	57 | 22 | 9 | 18
South 2008	554 | 87 | 35	390 | 158 | 14	473 | 17 | 2	440 | 15 | 9	375 | 44 | 50	310 | 168 | 25	428 | 15 | 6	374 | 15 | 10	348 | 254 | 51 | 67
South 2013	774 | 144 | 17	455 | 289 | 16	642 | 74 | 8	606 | 58 | 8	539 | 56 | 48	439 | 250 | 15	618 | 59 | 8	583 | 56 | 9	437 | 454 | 48 | 51
South 2018	822 | 95 | 29	589 | 41 | 9	637 | 7 | 2	637 | 12 | 4	516 | 22 | 37	583 | 42 | 7	620 | 8 | 1	624 | 13 | 11	730 | 148 | 21 | 62

Calculations use crude measures of MOV. Dose columns show counts: Vaccinated at First Eligible Opportunity | MOV - Later Corrected | MOV - Uncorrected.

Summary column shows, for each stratum, counts: No MOVs | All MOVs Corrected | Mixture Corrected/Uncorrected | All MOVs Uncorrected.
Summary column calculated using the following doses: BCG, DPT1, DPT2, DPT3, MCV1, OPV1, OPV2, OPV3

Table SC.3a. Child-based MOSV analysis – Colombia – National Results

Stratum	BCG	DPT1	DPT2	DPT3	MCV	OPV1	OPV2	OPV3	Summary
Colombia 1995	469 | 85 | 23	452 | 39 | 3	337 | 8 | 6	275 | 20 | 3	51 | 4 | 6	457 | 35 | 4	342 | 12 | 8	279 | 18 | 5	436 | 139 | 15 | 29
Colombia 2000	457 | 168 | 41	487 | 118 | 9	409 | 92 | 9	375 | 81 | 17	347 | 51 | 41	492 | 113 | 8	424 | 77 | 12	360 | 77 | 21	232 | 326 | 72 | 53
Colombia 2005	1788 | 290 | 50	1912 | 82 | 19	1625 | 57 | 9	1430 | 85 | 38	1340 | 80 | 58	1740 | 126 | 162	1509 | 60 | 11	1321 | 71 | 40	1398 | 479 | 92 | 235
Colombia 2010	2510 | 141 | 40	2427 | 59 | 22	2402 | 35 | 8	2213 | 50 | 21	1990 | 80 | 68	2415 | 76 | 62	2388 | 41 | 10	2205 | 47 | 37	2156 | 363 | 38 | 193

Calculations use crude measures of MOV. Dose columns show counts: Vaccinated at First Eligible Opportunity | MOV - Later Corrected | MOV - Uncorrected.

Summary column shows, for each stratum, counts: No MOVs | All MOVs Corrected | Mixture Corrected/Uncorrected | All MOVs Uncorrected.
Summary column calculated using the following doses: BCG, DPT1, DPT2, DPT3, MCV, OPV1, OPV2, OPV3

Figure SC.3. Child-based MOSV analysis – Colombia - Subnational Detail

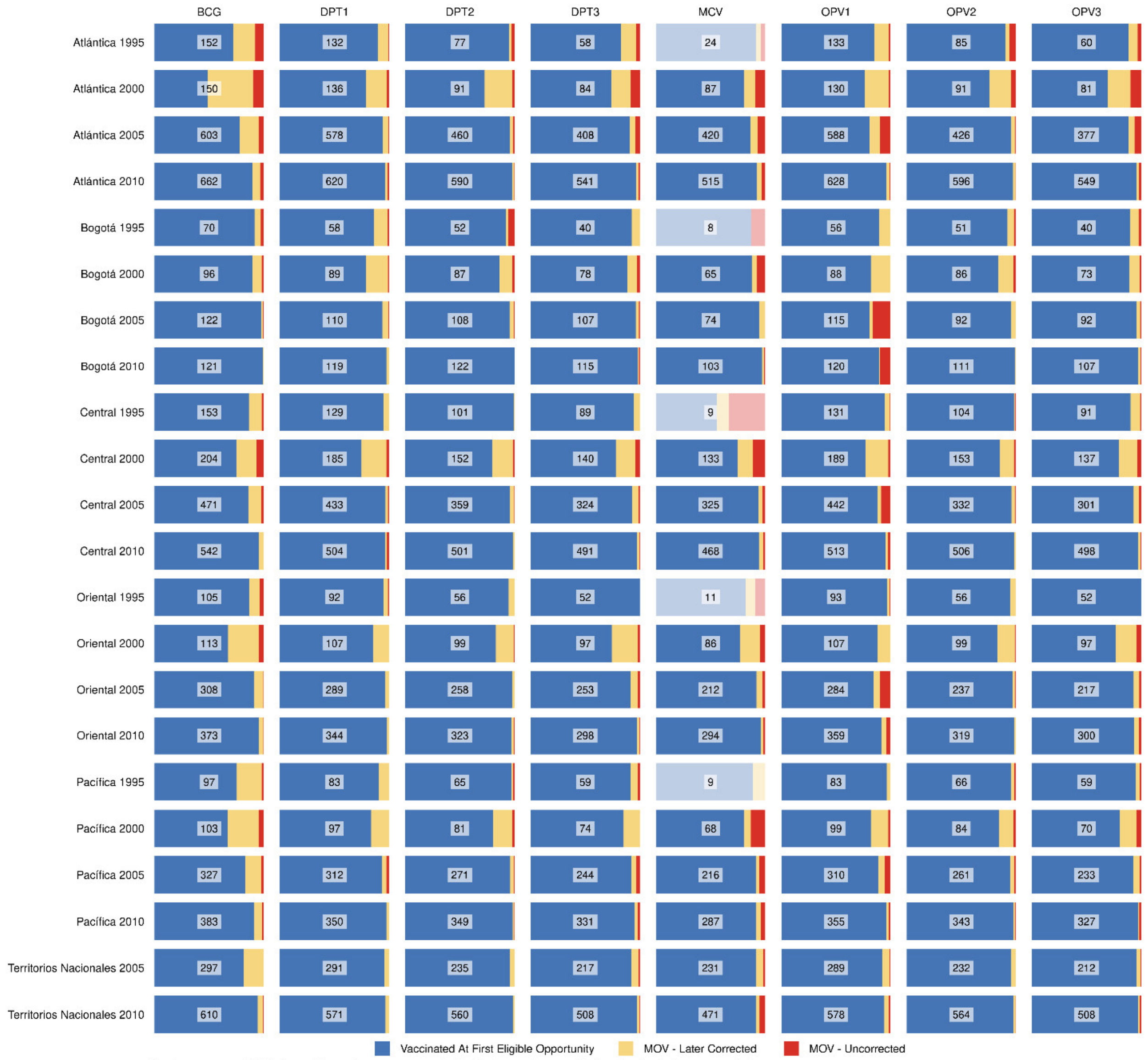

Crude measures of MOV shown. Figure shows suppressed output for bars where n < 25. Sample size is shown on each bar.

The stratum summary table shows information for the following doses: BCG, DPT1, DPT2, DPT3, MCV, OPV1, OPV2, OPV3.

Stratum	NM	AC	SC	NC
Atlántica 1995	60.9	26.6	4.7	7.7
Atlántica 2000	23.9	51.0	12.9	12.3
Atlántica 2005	54.3	24.8	7.3	13.6
Atlántica 2010	76.4	14.8	1.8	7.0
Bogotá 1995	68.0	24.0	1.3	6.7
Bogotá 2000	53.6	35.1	6.2	5.2
Bogotá 2005	69.6	12.0	3.2	15.2
Bogotá 2010	83.1	6.5	0.8	9.7
Central 1995	77.4	18.3	2.4	1.8
Central 2000	31.1	48.1	13.1	7.8
Central 2005	66.6	19.0	3.1	11.3
Central 2010	81.1	12.3	0.7	5.9
Oriental 1995	80.6	14.8	0.0	4.6
Oriental 2000	36.8	51.3	7.7	4.3
Oriental 2005	67.8	19.2	1.9	11.0
Oriental 2010	77.9	14.2	1.3	6.6
Pacífica 1995	66.0	29.1	1.9	2.9
Pacífica 2000	33.3	50.0	9.3	7.4
Pacífica 2005	62.9	21.9	4.4	10.8
Pacífica 2010	76.4	14.7	1.0	7.9
Territorios Nacionales 2005	70.5	26.0	1.9	1.6
Territorios Nacionales 2010	78.9	12.0	1.9	7.1

MOV Summary by Stratum. % kids with:
NM = no MOVs; AC = all MOVs corrected;
SC = some corrected; NC = none corrected.
Percentages in each row sum to 100%.

Table SC.3b. Child-based MOSV analysis – Colombia - Subnational Detail

Stratum	BCG	DPT1	DPT2	DPT3	MCV	OPV1	OPV2	OPV3	Summary
Atlántica 1995	109 | 31 | 12	118 | 13 | 1	73 | 2 | 2	48 | 8 | 2	22 | 1 | 1	113 | 18 | 2	77 | 3 | 5	53 | 5 | 2	103 | 45 | 8 | 13
Atlántica 2000	73 | 62 | 15	107 | 26 | 3	66 | 23 | 2	62 | 15 | 7	70 | 9 | 8	99 | 29 | 2	69 | 18 | 4	56 | 17 | 8	37 | 79 | 20 | 19
Atlántica 2005	471 | 103 | 29	543 | 31 | 4	440 | 15 | 5	370 | 22 | 16	362 | 29 | 29	476 | 54 | 58	406 | 17 | 3	332 | 22 | 23	340 | 155 | 46 | 85
Atlántica 2010	593 | 46 | 23	596 | 15 | 9	577 | 11 | 2	523 | 11 | 7	475 | 23 | 17	602 | 19 | 7	580 | 13 | 3	526 | 12 | 11	520 | 101 | 12 | 48
Bogotá 1995	64 | 4 | 2	50 | 7 | 1	48 | 1 | 3	37 | 3 | 0	7 | 0 | 1	50 | 6 | 0	47 | 3 | 1	36 | 3 | 1	51 | 18 | 1 | 5
Bogotá 2000	86 | 8 | 2	70 | 18 | 1	75 | 10 | 2	69 | 7 | 2	57 | 3 | 5	72 | 16 | 0	72 | 12 | 2	65 | 7 | 1	52 | 34 | 6 | 5
Bogotá 2005	119 | 2 | 1	103 | 6 | 1	103 | 4 | 1	103 | 3 | 1	70 | 4 | 0	93 | 3 | 19	88 | 4 | 0	88 | 3 | 1	87 | 15 | 4 | 19
Bogotá 2010	120 | 1 | 0	116 | 3 | 0	122 | 0 | 0	113 | 1 | 1	100 | 2 | 1	107 | 1 | 12	110 | 1 | 0	104 | 2 | 1	103 | 8 | 1 | 12
Central 1995	132 | 18 | 3	122 | 7 | 0	100 | 1 | 0	84 | 5 | 0	5 | 1 | 3	124 | 6 | 1	102 | 1 | 1	82 | 8 | 1	127 | 30 | 4 | 3
Central 2000	153 | 37 | 14	138 | 42 | 5	121 | 29 | 2	109 | 25 | 6	99 | 19 | 15	146 | 39 | 4	130 | 20 | 3	109 | 23 | 5	64 | 99 | 27 | 16
Central 2005	404 | 57 | 10	418 | 10 | 5	344 | 14 | 1	301 | 18 | 5	305 | 12 | 8	390 | 14 | 38	318 | 12 | 2	280 | 15 | 6	319 | 91 | 15 | 54
Central 2010	517 | 23 | 2	486 | 8 | 10	494 | 6 | 1	478 | 10 | 3	441 | 19 | 8	490 | 11 | 12	498 | 7 | 1	484 | 10 | 4	450 | 68 | 4 | 33
Oriental 1995	91 | 10 | 4	87 | 4 | 1	53 | 3 | 0	52 | 0 | 0	9 | 1 | 1	90 | 2 | 1	53 | 3 | 0	52 | 0 | 0	87 | 16 | 0 | 5
Oriental 2000	76 | 32 | 5	91 | 16 | 0	82 | 16 | 1	72 | 23 | 2	66 | 16 | 4	94 | 13 | 0	82 | 16 | 1	74 | 19 | 4	43 | 60 | 9 | 5
Oriental 2005	281 | 25 | 2	278 | 10 | 1	253 | 5 | 0	231 | 17 | 5	195 | 12 | 5	240 | 16 | 28	229 | 6 | 2	202 | 11 | 4	215 | 61 | 6 | 35
Oriental 2010	356 | 15 | 2	337 | 7 | 0	314 | 6 | 3	289 | 8 | 1	282 | 7 | 5	330 | 15 | 14	314 | 4 | 1	280 | 13 | 7	296 | 54 | 5 | 25
Pacífica 1995	73 | 22 | 2	75 | 8 | 0	63 | 1 | 1	54 | 4 | 1	8 | 1 | 0	80 | 3 | 0	63 | 2 | 1	56 | 2 | 1	68 | 30 | 2 | 3
Pacífica 2000	69 | 29 | 5	81 | 16 | 0	65 | 14 | 2	63 | 11 | 0	55 | 4 | 9	81 | 16 | 2	71 | 11 | 2	56 | 11 | 3	36 | 54 | 10 | 8
Pacífica 2005	271 | 48 | 8	292 | 12 | 8	260 | 9 | 2	225 | 11 | 8	197 | 7 | 12	275 | 19 | 16	247 | 10 | 4	216 | 13 | 4	215 | 75 | 15 | 37
Pacífica 2010	349 | 28 | 6	341 | 8 | 1	343 | 5 | 1	315 | 9 | 7	262 | 13 | 12	342 | 7 | 6	335 | 6 | 2	317 | 4 | 6	301 | 58 | 4 | 31
Territorios Nacionales 2005	242 | 55 | 0	278 | 13 | 0	225 | 10 | 0	200 | 14 | 3	211 | 16 | 4	266 | 20 | 3	221 | 11 | 0	203 | 7 | 2	222 | 82 | 6 | 5
Territorios Nacionales 2010	575 | 28 | 7	551 | 18 | 2	552 | 7 | 1	495 | 11 | 2	430 | 16 | 25	544 | 23 | 11	551 | 10 | 3	494 | 6 | 8	486 | 74 | 12 | 44

Calculations use crude measures of MOV. Dose columns show counts: Vaccinated at First Eligible Opportunity | MOV - Later Corrected | MOV - Uncorrected.

Summary column shows, for each stratum, counts: No MOVs | All MOVs Corrected | Mixture Corrected/Uncorrected | All MOVs Uncorrected.
Summary column calculated using the following doses: BCG, DPT1, DPT2, DPT3, MCV, OPV1, OPV2, OPV3

Table SN.4. MOSV Consequences – Nigeria – Potential Coverage Increase – National Results

	Card Seen (%)	Crude Cvg (%)	Up (%)	Valid Cvg (%)	Up (%)	Crude Cvg (%)	Up (%)	Valid Cvg (%)	Up (%)	Crude Cvg (%)	Up (%)	Valid Cvg (%)	Up (%)	Crude Cvg (%)	Up (%)	Valid Cvg (%)	Up (%)
		BCG				OPV1				OPV2				OPV3			
1990	36	61	1	34	2	64	1	33	2	48	0	22	1	35	0	13	3
1999	21	57	1	19	1	59	1	17	3	48	2	13	5	28	4	7	8
2003	22	49	1	20	1	67	3	16	4	54	3	10	5	31	2	5	6
2008	26	50	2	21	5	68	2	21	4	58	2	16	5	39	2	9	8
2013	28	51	1	24	3	77	1	24	2	70	1	20	4	54	1	11	10
2018	40	67	2	35	3	74	1	34	2	67	1	29	3	48	1	17	11

	Card Seen (%)	Crude Cvg (%)	Up (%)	Valid Cvg (%)	Up (%)	Crude Cvg (%)	Up (%)	Valid Cvg (%)	Up (%)	Crude Cvg (%)	Up (%)	Valid Cvg (%)	Up (%)	Crude Cvg (%)	Up (%)	Valid Cvg (%)	Up (%)
		DPT1				DPT2				DPT3				MCV1			
1990	36	63	1	32	2	48	0	22	1	34	0	13	4	45	4	19	5
1999	21	50	3	16	3	42	3	12	6	29	4	7	8	43	1	10	2
2003	22	43	3	17	3	33	2	11	4	23	2	5	6	37	2	9	2
2008	26	52	1	23	2	45	1	18	3	36	1	11	6	41	2	14	2
2013	28	51	1	25	2	46	1	21	3	39	1	12	8	42	2	15	3
2018	40	65	1	34	2	58	1	30	3	51	1	17	10	54	2	18	3

Gold columns labeled 'Up (%)' show increase in crude or valid coverage if respondents with cards had received every eligible dose at every documented vaccination visit.

Color bars are scaled so that 100% would fill the entire table cell.

All outcomes are weighted.

Table SC.4. MOSV Consequences – Colombia – Potential Coverage Increase – National Results

	Card Seen (%)	Crude Cvg (%)	Up (%)	Valid Cvg (%)	Up (%)	Crude Cvg (%)	Up (%)	Valid Cvg (%)	Up (%)	Crude Cvg (%)	Up (%)	Valid Cvg (%)	Up (%)	Crude Cvg (%)	Up (%)	Valid Cvg (%)	Up (%)
		BCG				OPV1				OPV2				OPV3			
1995	62	94	2	54	7	97	0	59	1	91	1	56	3	77	1	40	11
2000	75	95	3	69	6	97	1	73	2	89	2	67	4	72	5	54	13
2005	78	97	2	74	4	89	7	71	7	83	7	68	8	70	8	56	15
2010	83	97	1	75	3	94	3	75	3	91	3	74	4	83	4	65	9

	Card Seen (%)	Crude Cvg (%)	Up (%)	Valid Cvg (%)	Up (%)	Crude Cvg (%)	Up (%)	Valid Cvg (%)	Up (%)	Crude Cvg (%)	Up (%)	Valid Cvg (%)	Up (%)	Crude Cvg (%)	Up (%)	Valid Cvg (%)	Up (%)
		DPT1				DPT2				DPT3				MCV1			
1995	62	96	0	59	1	88	1	55	3	78	1	40	10	84	1	5	3
2000	75	95	1	72	2	89	2	67	4	77	4	54	13	71	4	44	7
2005	78	97	1	77	1	89	1	74	2	82	2	61	10	82	2	50	7
2010	83	97	1	77	1	94	1	75	2	90	2	65	9	81	1	59	3

Gold columns labeled 'Up (%)' show increase in crude or valid coverage if respondents with cards had received every eligible dose at every documented vaccination visit.

Color bars are scaled so that 100% would fill the entire table cell.

All outcomes are weighted.

Table SN.5a. MOSV Consequences – Nigeria – Time to Correction – National Results

levelname	BCG	DPT1	DPT2	DPT3	MCV1	OPV1	OPV2	OPV3
Nigeria 1990	4	27	30	133	28	35	52	109	30	31	82	113	1	1	1	1	32	61	118	138	28	35	50	78	24	29	149	221	4	6	16	22
Nigeria 1999	27	38	169	300	28	35	55	108	3	4	5	6	11	12	14	14	39	91	140	188	28	35	56	78	3	20	87	163	182	182	182	182
Nigeria 2003	6	58	148	192	28	42	70	165	6	8	18	24	23	40	52	209	30	50	158	203	28	36	62	102	11	19	27	32	54	61	213	304
Nigeria 2008	15	45	110	190	28	35	62	109	14	31	87	150	28	41	94	140	34	68	120	194	28	40	68	118	28	40	95	129	28	32	75	167
Nigeria 2013	10	28	63	154	27	35	67	133	20	36	66	133	26	44	90	155	28	40	92	163	28	35	59	111	21	35	66	131	28	34	83	158
Nigeria 2018	7	21	48	119	27	34	55	91	3	8	28	38	7	20	126	238	32	71	116	187	27	34	50	86	4	10	28	34	6	14	60	190

Table shows 25th, 50th, 75th, and 90th percentiles for days between first MOV and receiving dose, by level and dose.

Table SN.5b. MOSV Consequences – Nigeria – Time to Correction – Subnational Detail

levelname	BCG	DPT1	DPT2	DPT3	MCV1	OPV1	OPV2	OPV3
Centr 1999	30 | 104 | 187 | 220	31 | 37 | 49 | 61	2 | 2 | 2 | 2	NA | NA | NA | NA	259 | 259 | 259 | 259	32 | 42 | 58 | 84	NA | NA | NA | NA	NA | NA | NA | NA
North 1990	6 | 22 | 81 | 186	31 | 46 | 59 | 119	31 | 31 | 31 | 31	NA | NA | NA | NA	38 | 63 | 113 | 129	30 | 42 | 52 | 63	24 | 29 | 149 | 221	6 | 6 | 6 | 6
North 1999	16 | 31 | 61 | 79	18 | 30 | 33 | 44	3 | 3 | 3 | 3	15 | 15 | 15 | 15	91 | 138 | 142 | 170	22 | 44 | 67 | 78	NA | NA | NA | NA	NA | NA | NA | NA
North 2003	7 | 32 | 148 | 164	28 | 42 | 77 | 198	13 | 18 | 23 | 26	24 | 32 | 42 | 49	30 | 50 | 105 | 193	26 | 42 | 62 | 113	3 | 3 | 3 | 3	61 | 61 | 61 | 61
North 2008	28 | 63 | 135 | 229	28 | 42 | 76 | 161	26 | 36 | 88 | 147	28 | 35 | 64 | 124	34 | 63 | 110 | 157	28 | 42 | 76 | 159	28 | 35 | 92 | 133	31 | 46 | 118 | 198
North 2013	17 | 42 | 77 | 176	25 | 33 | 68 | 147	28 | 43 | 82 | 143	28 | 42 | 68 | 159	25 | 40 | 102 | 181	28 | 35 | 65 | 132	19 | 42 | 87 | 135	27 | 28 | 60 | 153
North 2018	7 | 28 | 58 | 128	28 | 35 | 56 | 90	4 | 12 | 28 | 35	8 | 14 | 154 | 365	30 | 63 | 115 | 188	28 | 35 | 56 | 88	4 | 10 | 27 | 44	6 | 10 | 42 | 254
South 1990	15 | 27 | 28 | 28	28 | 35 | 48 | 91	56 | 82 | 108 | 124	1 | 1 | 1 | 1	30 | 60 | 117 | 139	28 | 35 | 50 | 99	NA | NA | NA | NA	7 | 14 | 20 | 24
South 1999	29 | 46 | 250 | 428	28 | 35 | 56 | 143	5 | 6 | 6 | 6	10 | 10 | 10 | 10	21 | 22 | 56 | 79	28 | 35 | 55 | 77	3 | 20 | 87 | 163	182 | 182 | 182 | 182
South 2003	44 | 84 | 163 | 210	28 | 35 | 62 | 154	5 | 5 | 5 | 5	34 | 47 | 206 | 301	72 | 115 | 158 | 185	28 | 35 | 62 | 81	35 | 35 | 35 | 35	126 | 206 | 286 | 333
South 2008	12 | 32 | 80 | 142	28 | 34 | 55 | 83	7 | 28 | 84 | 142	28 | 65 | 118 | 183	36 | 71 | 135 | 195	28 | 36 | 64 | 97	28 | 49 | 96 | 117	28 | 28 | 34 | 77
South 2013	7 | 21 | 48 | 137	28 | 35 | 63 | 118	15 | 34 | 62 | 76	24 | 47 | 92 | 149	29 | 39 | 90 | 155	28 | 34 | 56 | 93	23 | 35 | 62 | 126	28 | 39 | 91 | 168
South 2018	7 | 14 | 28 | 62	14 | 28 | 42 | 105	2 | 3 | 12 | 48	6 | 47 | 94 | 129	40 | 86 | 117 | 181	16 | 28 | 42 | 63	6 | 24 | 28 | 30	7 | 35 | 60 | 118

Table shows 25th, 50th, 75th, and 90th percentiles for days between first MOV and receiving dose, by level and dose.

Table SC.5a. MOSV Consequences – Colombia – Potential Coverage Increase – National Results

levelname	BCG	DPT1	DPT2	DPT3	MCV	OPV1	OPV2	OPV3
Colombia 1995	20 | 63 | 152 | 253	14 | 30 | 42 | 167	34 | 53 | 83 | 155	14 | 28 | 71 | 128	24 | 38 | 58 | 71	29 | 33 | 63 | 146	9 | 21 | 36 | 59	16 | 29 | 77 | 128
Colombia 2000	10 | 36 | 103 | 181	10 | 35 | 70 | 132	16 | 40 | 80 | 163	20 | 47 | 86 | 181	60 | 98 | 170 | 187	13 | 41 | 70 | 130	12 | 30 | 63 | 141	22 | 40 | 98 | 168
Colombia 2005	6 | 21 | 70 | 146	9 | 31 | 59 | 81	6 | 22 | 59 | 99	6 | 28 | 62 | 181	9 | 30 | 65 | 155	15 | 40 | 67 | 114	5 | 22 | 58 | 117	6 | 30 | 63 | 195
Colombia 2010	4 | 26 | 61 | 103	8 | 32 | 61 | 96	3 | 7 | 31 | 55	6 | 30 | 66 | 178	22 | 39 | 100 | 191	10 | 33 | 61 | 86	1 | 5 | 27 | 39	10 | 30 | 64 | 178

Table shows 25th, 50th, 75th, and 90th percentiles for days between first MOV and receiving dose, by level and dose.

Table SC.5b. MOSV Consequences – Colombia – Potential Coverage Increase – Subnational Detail

levelname	BCG	DPT1	DPT2	DPT3	MCV	OPV1	OPV2	OPV3
Atlántica 1995	26 | 72 | 150 | 195	20 | 30 | 61 | 206	72 | 93 | 114 | 127	8 | 29 | 62 | 146	24 | 24 | 24 | 24	28 | 33 | 59 | 151	10 | 15 | 68 | 100	27 | 38 | 102 | 143
Atlántica 2000	7 | 30 | 134 | 213	9 | 29 | 59 | 81	3 | 19 | 43 | 61	6 | 17 | 58 | 74	64 | 74 | 174 | 176	9 | 42 | 78 | 104	6 | 18 | 70 | 161	7 | 20 | 59 | 107
Atlántica 2005	7 | 30 | 82 | 164	15 | 30 | 54 | 81	12 | 30 | 72 | 117	6 | 28 | 62 | 153	7 | 30 | 65 | 158	22 | 45 | 66 | 101	4 | 22 | 70 | 136	6 | 24 | 93 | 228
Atlántica 2010	7 | 26 | 50 | 102	6 | 35 | 56 | 62	8 | 20 | 34 | 82	18 | 34 | 119 | 153	19 | 34 | 69 | 141	6 | 31 | 56 | 62	3 | 4 | 28 | 36	16 | 32 | 90 | 185
Bogotá 1995	18 | 26 | 94 | 208	8 | 32 | 33 | 37	27 | 27 | 27 | 27	10 | 16 | 17 | 18	NA | NA | NA | NA	32 | 38 | 70 | 90	6 | 6 | 16 | 23	10 | 16 | 17 | 18
Bogotá 2000	10 | 22 | 29 | 124	31 | 57 | 69 | 136	37 | 80 | 116 | 205	36 | 40 | 54 | 67	104 | 142 | 206 | 244	32 | 65 | 72 | 148	30 | 58 | 92 | 192	40 | 55 | 72 | 119
Bogotá 2005	6 | 8 | 9 | 10	8 | 29 | 31 | 58	4 | 6 | 17 | 36	19 | 30 | 46 | 56	25 | 92 | 160 | 173	15 | 28 | 30 | 30	4 | 6 | 14 | 28	4 | 8 | 19 | 26
Bogotá 2010	1 | 1 | 1 | 1	33 | 63 | 66 | 69	NA | NA | NA | NA	245 | 245 | 245 | 245	133 | 158 | 182 | 197	63 | 63 | 63 | 63	1 | 1 | 1 | 1	10 | 10 | 10 | 10
Central 1995	26 | 76 | 128 | 246	24 | 42 | 144 | 215	55 | 55 | 55 | 55	28 | 30 | 65 | 79	24 | 24 | 24 | 24	24 | 38 | 110 | 164	28 | 28 | 28 | 28	16 | 29 | 50 | 130
Central 2000	13 | 37 | 88 | 219	10 | 33 | 103 | 154	21 | 38 | 64 | 128	32 | 56 | 129 | 164	86 | 134 | 181 | 187	10 | 36 | 66 | 137	18 | 33 | 62 | 119	34 | 63 | 126 | 178
Central 2005	7 | 19 | 63 | 151	3 | 20 | 56 | 59	12 | 39 | 68 | 95	3 | 8 | 35 | 69	21 | 32 | 61 | 68	35 | 60 | 86 | 198	4 | 15 | 60 | 94	6 | 28 | 36 | 63
Central 2010	6 | 40 | 68 | 167	25 | 61 | 90 | 262	2 | 4 | 6 | 8	8 | 30 | 42 | 144	28 | 69 | 110 | 182	25 | 32 | 61 | 64	2 | 6 | 14 | 26	4 | 9 | 40 | 175
Oriental 1995	7 | 16 | 99 | 177	2 | 8 | 24 | 43	25 | 36 | 120 | 170	NA | NA | NA | NA	51 | 51 | 51 | 51	24 | 35 | 46 | 52	12 | 14 | 25 | 32	NA | NA | NA | NA
Oriental 2000	13 | 40 | 103 | 160	9 | 38 | 56 | 82	15 | 29 | 80 | 156	24 | 47 | 85 | 173	50 | 96 | 134 | 176	10 | 36 | 66 | 90	13 | 26 | 62 | 132	15 | 31 | 116 | 145
Oriental 2005	5 | 25 | 70 | 134	11 | 33 | 60 | 94	1 | 12 | 15 | 23	10 | 30 | 179 | 231	28 | 46 | 99 | 175	8 | 30 | 40 | 92	18 | 28 | 95 | 117	28 | 35 | 80 | 184
Oriental 2010	1 | 4 | 55 | 135	20 | 30 | 49 | 84	6 | 22 | 58 | 215	1 | 6 | 53 | 138	4 | 31 | 120 | 160	30 | 58 | 62 | 62	2 | 6 | 14 | 22	4 | 48 | 61 | 160
Pacífica 1995	36 | 52 | 176 | 252	25 | 30 | 32 | 67	66 | 66 | 66 | 66	20 | 68 | 114 | 122	80 | 80 | 80 | 80	30 | 31 | 88 | 122	42 | 48 | 55 | 58	53 | 72 | 91 | 102
Pacífica 2000	17 | 58 | 85 | 128	20 | 43 | 80 | 122	30 | 76 | 152 | 197	57 | 92 | 184 | 272	35 | 68 | 114 | 156	32 | 55 | 78 | 150	12 | 19 | 38 | 60	32 | 84 | 136 | 180
Pacífica 2005	5 | 28 | 80 | 148	25 | 40 | 63 | 116	5 | 22 | 59 | 111	10 | 30 | 49 | 89	6 | 26 | 34 | 50	34 | 62 | 70 | 122	14 | 38 | 53 | 67	9 | 30 | 69 | 174
Pacífica 2010	4 | 22 | 58 | 111	12 | 38 | 59 | 71	7 | 10 | 28 | 35	2 | 9 | 10 | 49	22 | 31 | 53 | 115	6 | 15 | 38 | 63	5 | 18 | 37 | 42	20 | 36 | 51 | 57
Territorios Nacionales 2005	4 | 11 | 33 | 76	5 | 31 | 48 | 62	10 | 20 | 30 | 113	6 | 43 | 86 | 117	6 | 16 | 64 | 94	6 | 30 | 60 | 93	3 | 15 | 38 | 88	6 | 9 | 51 | 107
Territorios Nacionales 2010	8 | 30 | 63 | 84	7 | 30 | 61 | 89	1 | 1 | 4 | 16	11 | 58 | 113 | 174	31 | 58 | 198 | 230	4 | 32 | 76 | 111	1 | 2 | 10 | 161	39 | 79 | 120 | 150

Table shows 25th, 50th, 75th, and 90th percentiles for days between first MOV and receiving dose, by level and dose.
